# Controlled Annealing in Adaptive Multicomponent Gels

**DOI:** 10.1002/anie.202215813

**Published:** 2022-12-14

**Authors:** Paolo Ravarino, Santanu Panja, Simona Bianco, Todor Koev, Matthew Wallace, Dave J. Adams

**Affiliations:** ^1^ Dipartimento di Chimica Giacomo Ciamician Alma Mater Studiorum Università di Bologna Via Selmi, 2 40126 Bologna Italy; ^2^ School of Chemistry University of Glasgow Glasgow G12 8QQ UK; ^3^ School of Pharmacy University of East Anglia Norwich Research Park Norwich NR4 7TJ UK

**Keywords:** Annealing, Co-Assembly, Multicomponent Hydrogel, Rheology

## Abstract

We use a pH‐driven annealing process to convert between co‐assembled and self‐sorted networks in multicomponent gels. The initially formed gels at low pH are co‐assembled, with the two components coexisting within the same self‐assembled structures. We use an enzymatic approach to increase the pH, resulting in a gel‐to‐sol transition, followed by a hydrolysis to lower the pH once again. As the pH decreases, a self‐sorted network is formed by a two‐stage gelation process determined by the p*K*_a_ of each component. This approach can be expanded to layered systems to generate many varied systems by changing composition and rates of pH change, adapting their microstructure and so allowing access to a far greater range of morphologies and complexity than can be achieved in single component systems.

## Graphical abstract

Annealing changes the assembly of fibres and leads to self‐sorting in the case of multicomponent systems. When a multilayer gel is formed, annealing can be used to form hybrid materials.

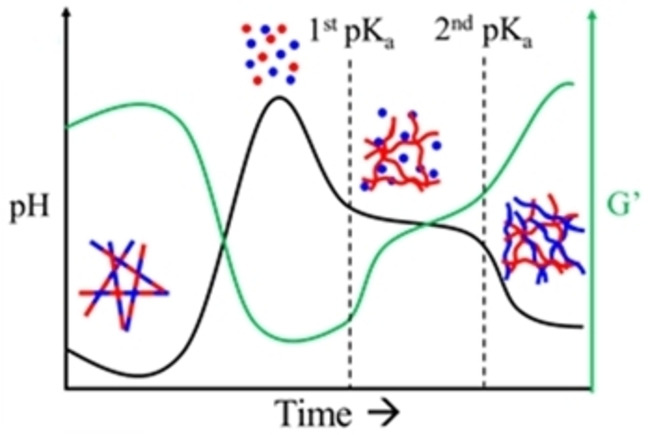

There is significant current interest in systems that evolve and change with time,^[1]^ both from a fundamental perspective and from that of mimicking life. There are many examples where self‐assembled systems are pre‐programmed to change their phase with time, for example from a solution to a gel to a solution once again. The times for the transitions can be controlled by the presence of additives designed to (for example) change the pH in a predetermined manner.

Most current examples focus on a single self‐assembling component. This component self‐assembles in different ways controlled by the solution conditions. Hence, the changes in the global system control the self‐assembled structures present and hence changes in, for example pH, result directly in whether structures such as micelles or persistent fibers are formed, the latter of which would typically lead to a gel phase.^[2]^ These systems are elegant. However, there are limited options when using single component systems.

In terms of multicomponent systems, the situation is more complex. Where both components can adopt different self‐assembled structures, it is possible to form structures which comprise either both or either of the components to give co‐assembled or self‐sorted systems respectively (shown schematically in Figure 1d).^[3]^ Conceptually this could change depending on the exact global conditions, providing an opportunity to generate extremely complex systems from a small number of components. For example, Nakamura et al. have shown how to use an out‐of‐equilibrium approach to form patterns in a self‐sorted network.^[4]^ Singh et al. have recently described the use of a fuel‐driven approach to form a three‐component, self‐sorted system.^[5]^

**Figure 1:**
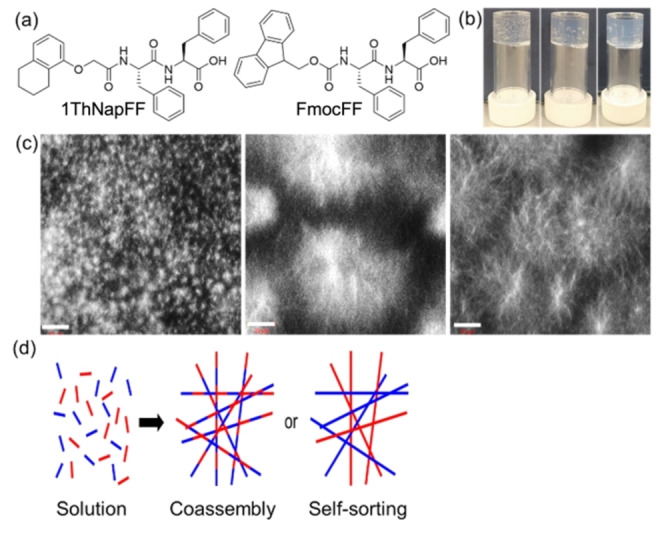
(a) Chemical structures of 1ThNapFF and FmocFF; (b) From left to right photographs of the gels obtained with 1ThNapFF, FmocFF and the multicomponent system (1ThNapFF+FmocFF); (c) From left to right: confocal microscope images of the gels of 1ThNapFF, FmocFF and the multicomponent system. Scale bars are 20 μm; (d) Cartoon showing co‐assembly and self‐sorting of the fibers in multicomponent gels.

Here, we describe a two‐component system formed from two dipeptides. A number of ways have been described to control pH changes in aqueous systems.^[6]^ Here, coupling with the well‐established urease‐urea^[7]^ and methyl formate combination that drives the pH up and then down,^[8]^ these dipeptides form self‐assembled structures which can be controlled directly by the pH. This approach allows us to directly control what structures will be formed in such a complex, multicomponent system. We show this approach can be used in layered systems to provide complex multicomponent systems by changing composition and adapting microstructure depending on the annealing process used.

1ThNapFF^[8]^ and FmocFF^[9]^ (Figure 1a) both form gels when water is added to a solution of either dipeptide in DMSO (Figure 1b). At a solvent composition of 20 % DMSO/80 % water, the critical gelation concentrations for 1ThNapFF and FmocFF are 0.25 mg mL^−1^ and 0.5 mg mL^−1^ respectively. Adding water to a mixture of both 1ThNapFF and FmocFF in DMSO also results in a gel (Figure 1b). The pH of this gel as formed was around 4.5. Rheologically, the multicomponent gel is stiffer than the gels formed using 1ThNapFF or FmocFF alone, with a frequency independent storage modulus (G′) and loss modulus (G′′) (Figure S5–S7). This is expected as there is additional gelator within the system, although we highlight it is difficult to predict such effects in advance due to differences in supersaturation^[10]^ and microstructure within the multicomponent systems as compared to the single component systems. These gels are stable over extended periods of time with no apparent change.

Spherulitic domains of self‐assembled fibers are formed in all cases, resulting in a network that spans the sample and leads to a gel (Figure 1c). The spherulites are a result of phase separation on adding water to the DMSO solution of the gelator.^[11]^ The spherulites formed by 1ThNapFF are much smaller than those formed by FmocFF. For the multicomponent gels, the spherulites are intermediate in size and there is no evidence for the co‐existence of small spherulites and large spherulites formed by 1ThNapFF and FmocFF respectively. This is highly suggestive of co‐assembled structures being formed, although for such systems we highlight that the rate of gelation and supersaturation effects can also lead to changes in microstructure.[^10^, ^12^]

Similar gels are formed on addition of an aqueous solution containing urease, urea and methyl formate as described elsewhere for single component systems.^[8]^ The conversion of urea to ammonia and carbon dioxide results in an increase in pH from around 4.5 to around 8.2. At this pH, hydrolysis of methyl formate occurs, resulting in a decrease in pH once again. The rate of increase and decrease of pH can be predetermined by the concentrations of urease, urea and methyl formate used.[^7^, ^8^] As the pH increases, the gels formed from either the single components or from the mixture of 1ThNapFF and FmocFF change to a solution state. This is driven by the pH increasing above the apparent p*K*_a_ of the terminal carboxylic acid of the components (6.3 for 1ThNapFF and 7.4 for FmocFF in this solvent mixture, Figure S8) which results in an increase in solubility and a structural shift from fibrous structures to wormlike micellar structures.^[13]^ As the pH decreases again, each of the systems reforms a gel as reprotonation of the carboxylic acid occurs (Figure 2a and 2b). During this process, CO_2_ and ammonia are formed, which often results in gas bubbles being formed in the gels. We stress that these bubbles do not affect the reproducibility of the data discussed below.

**Figure 2:**
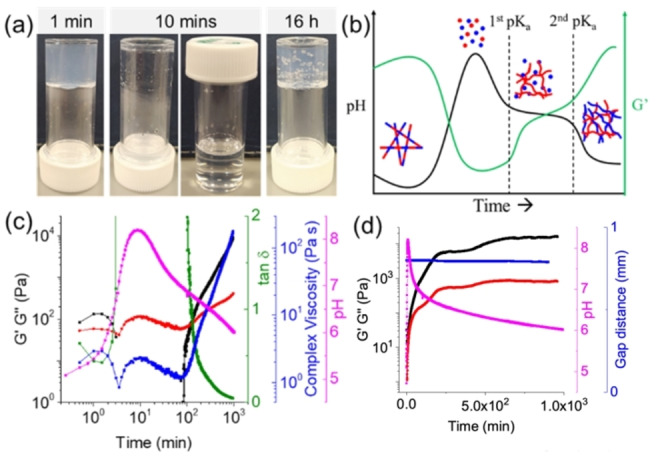
(a) Transition from gel to sol to gel with time for the multicomponent system in presence of urea‐urease and methyl formate; (b) Cartoon showing schematically the process as the pH increases and decreases in the multicomponent system – for clarity, we show the high pH situation as free molecules, but both systems actually form surfactant‐like aggregates; (c) Variation of pH (pink), G′ (black), G′′ (red), tanδ (green) and complex viscosity (blue) with time for the multicomponent system involving urea–urease and methyl formate reaction; A cup and vane system was used to collect the rheology data. (d) Variation of pH (pink), G′ (black), G′′ (red), and gap distance (blue) using parallel plates with time on a linear scale to show clearly the 2‐stage increase in G′ and G′′. For (a), (c) and (d), initial concentration of 1ThNapFF and FmocFF is 2 mg mL^−1^ for both, the concentration of urease is 0.4 mg mL^−1^, urea is 0.02 M and volume of methyl formate added is 0.82 M. The solvent is DMSO/H_2_O (20/80, v/v).

Following the entire process for the multicomponent system with time (Figure 2c) shows that the pH increases and decreases as expected. By rheology, G′ and G′′ are initially low, with G′ dominating over G′′. As the pH increases, G′ decreases, with G′′ increasing showing a liquid phase has been formed. G′ decreases in one stage as expected for a gel consisting of one network, in agreement with the assignment of a co‐assembled system. As the pH decreases again, G′ increases again to dominate over G′′ as a gel is reformed. This occurs below the apparent p*K*_a_ of FmocFF. There is an inflection in the rheology data when the apparent p*K*_a_ of 1ThNapFF is reached. Hence, the transition back to a gel phase occurs in a multistep fashion which can be linked directly to the apparent p*K*_a_ of each component.^[14]^ Indeed, we have shown previously that a difference in p*K*_a_ of one unit is sufficient for self‐sorting to occur in such systems.^[15]^ Carrying out the same experiment using a parallel plates geometry clearly shows the two‐stage increase in G′ and G′′ as the pH decreases once again (Figure 2d; note time here is on a linear scale). The constant gap distance that is maintained over the gelation process shows that these changes are not artefactual due to drying. Hence, we appear to have a system that starts as a co‐assembled system at low pH and can be annealed to form a self‐sorted system.

To confirm whether co‐assembly or self‐sorting are occurring in the initially formed and pH‐annealed systems, we used a number of techniques. Circular dichroism (CD) spectra (Figure 3a and 3b) are complicated by potential LD and possibly circular intensity differential scattering,^[16]^ but nonetheless show that the 1ThNapFF gels show similar data before and after pH annealing, whilst the FmocFF shows an inversion in sign around 230 nm on annealing. The multicomponent systems show differences before and after annealing between 250 and 300 nm. Whilst difficult to interpret, these data imply that there is a difference in packing in the multicomponent systems before and after the pH increase and decrease.

**Figure 3:**
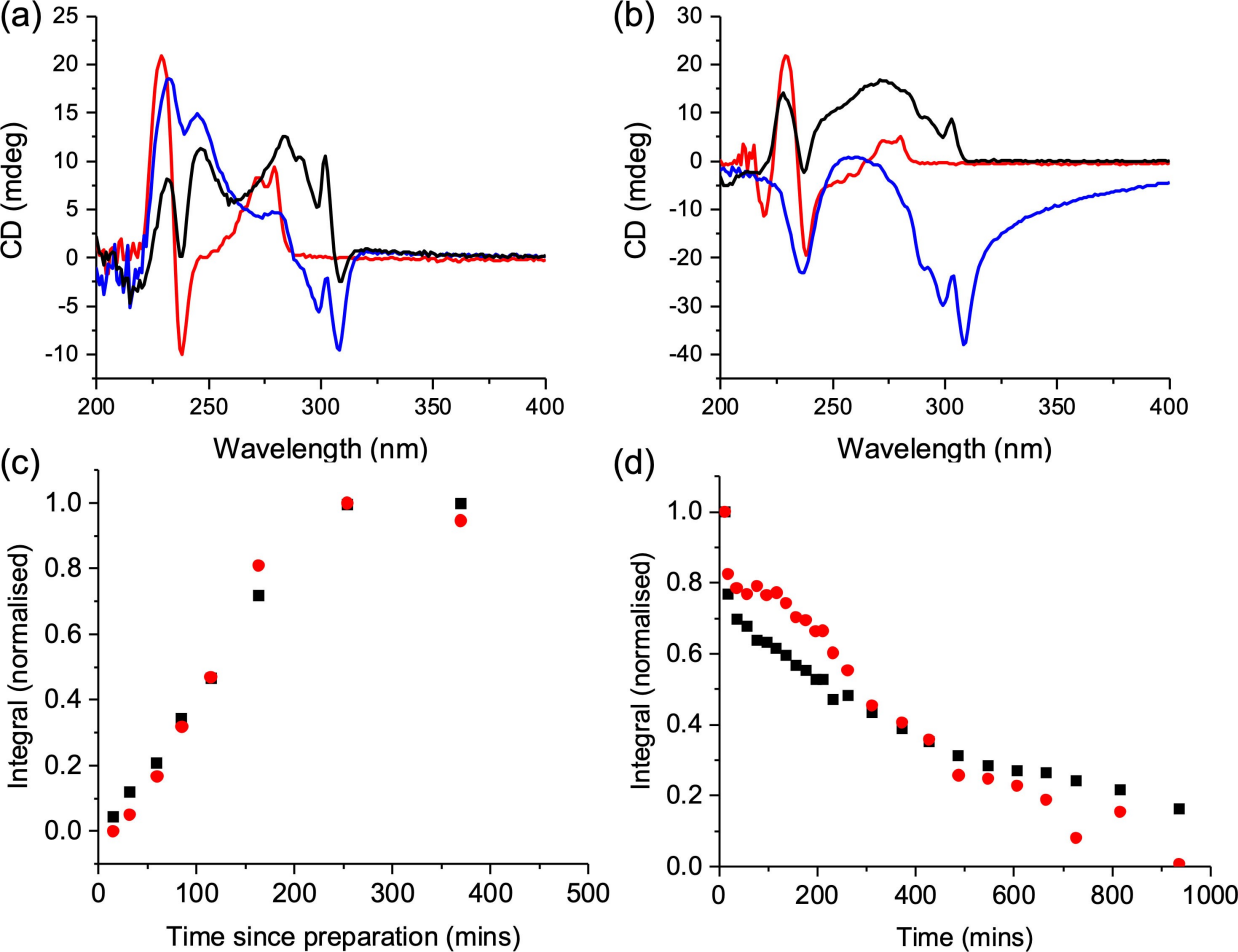
(a) CD spectra of the hydrogels of 1ThNapFF (red), FmocFF (blue) and the multicomponent system (black) as directly formed; (b) CD spectra of the hydrogels of 1ThNapFF (red), FmocFF (blue) and the multicomponent system (black) after the pH annealing. (c) Integrations from NMR spectra on going from low pH to high pH in presence of urea‐urease. (d) Integrations from NMR spectra on going from high pH to low pH in presence of urea‐urease and methyl formate. Integrals are plotted as integrals, normalised relative to the largest integral recorded in the data series. For (c) and (d) the black data are for the total aromatic region between 7.6 and 6.0 ppm and red data are for the cyclohexyl peaks on 1ThNapFF between 1.9 and 1.3 ppm.

^1^H NMR spectroscopy was used to follow the pH increase and pH decrease using the integrations of the gelators (Figure 3c and 3d; example spectra shown in Figure S9–S14). The fast rate of the initial pH increase (Figure 2c) precludes direct observation of this step by ^1^H NMR as it occurs before the sample can be loaded into the spectrometer. Therefore, to study the initial increase in pH, a separate sample was prepared without methyl formate at a reduced concentration of urease (10 μg mL^−1^), slowing down the rate of pH increase. Due to the broad resonances, it was not possible to integrate the FmocFF and 1ThNapFF separately (Figures S9–S11). However, the resonances of the 6 and 7 position of the cyclohexyl moiety of 1ThNapFF were resolvable. When the pH of a two‐component sample is increased with urea‐urease alone, the combined integral of the aromatic protons of both gelators, and the 6 and 7 position of 1ThNapFF increase at the same rate (Figure 3c), suggesting a single network is falling apart. Full spectra are provided in Figure S12. In comparison, as the pH decreases (Figure 3d), the integral of the aromatic protons of both gelators, and the cyclohexyl moiety of 1ThNapFF decrease at different rates, implying a self‐sorting behaviour in agreement with previous results.[^14^, ^15^] ^1^H integration indicates that essentially all (>50 %) of the gelators are soluble at the first time point in the urea‐urease methyl formate sample, although accurate integration is not possible due to the short relaxation delays necessary to capture the system at early time points when it is evolving rapidly (Figure S13). When the pH of a 2 mg mL^−1^ sample of 1ThNapFF alone decreases in the presence of urea‐urease and methyl formate, the aromatic and cyclohexyl protons decrease in intensity at the same rate (Figure S14). Overall, these data show that we initially have a co‐assembled network which dissolves as the pH increases and then a self‐sorted network is formed on pH decrease.

We can tune the rate of pH change (Figure S15) and hence the properties of the material (Figure S16) by varying the amounts of methyl formate, urea, or urease used. A decrease in the concentration of either urea or urease resulted in decrease in the rate of the pH increase during annealing. The rate of pH change can further be controlled by increasing the concentration of methyl formate. Since 1ThNapFF forms wormlike micelles at high pH^[13a]^ and there are indications that FmocFF does too,[^13b^, ^17^] the pH reached and the length of time spent at high pH will likely affect the aggregates formed, the charge on them (and hence persistence length etc.). Hence, even small changes in the rate of pH increase and decrease as well as differences in the time spent at the maximum pH will likely affect the mechanical properties of the gels formed on reacidification. Comparison of pH‐time profiles with the rheological behavior of the annealed gels shows that the mechanical properties of the final gels depend on the maximum pH during the pH cycles. A gradual decrease in maximum pH gives gels with a lower stiffness. All annealed gels show similar microstructure (Figure S17). It takes a significant time under these conditions for the gels to reach plateau values (Figure S18); in Figures S15–S17, we show data after 1000 minutes to ensure there are no drying artefacts in the comparison. We highlight that the gel properties will be controlled by factors such as the spherulites or other microstructure, number of crosslinks, and possible seeding of one network on another[^2e^, ^3c^, ^3d^, ^10^, ^12^, ^18^] and are essentially impossible to predict or tease apart in such a complicated multicomponent system. However, with control over the pH change, we get reproducible data showing that we can make gels with very similar properties time and time again and so the comparison at a set time is valid.

This annealing approach can be used to change the composition of a gel. For all of the discussion below, we highlight that we are using NMR data and rheological data to show that we can control the exact composition in a spatially resolved manner and that this leads to changes in the mechanical properties. The rheological properties evolve over long time periods (see Figure S18) and so we focus here on differences not on absolute values.

First, to exemplify the methodology, a layered gel was formed, with the bottom layer formed from only 1ThNapFF and the top layer from FmocFF. No urea, urease and methyl formate were included and so no annealing is expected. After 16 hours, the gel was sliced into 6 segments and the composition determined by NMR (Figure S19 and S20). As expected, after slicing into segments, each of the segments contained only 1ThNapFF or FmocFF (Figure 4a and 4b, Figure S21). Each section of the gel is as stiff as the section of the single layer gel prepared and analyzed in the same way as the corresponding gelator alone (Figure S22). NMR and rheology data are summarized together with confocal images of each section supporting the lack of mixing in Figure 4a–d.

**Figure 4:**
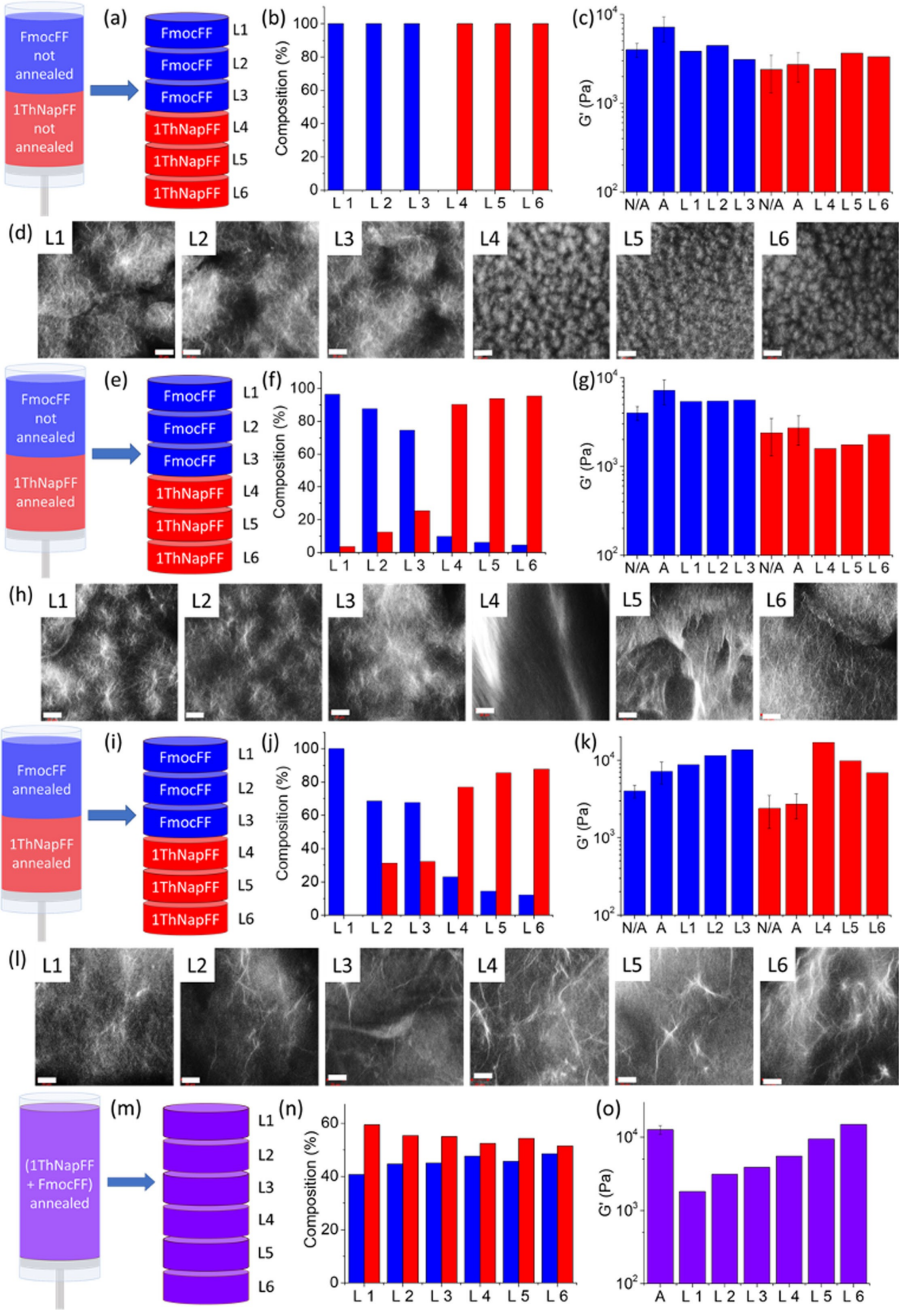
Experiments with multilayer gels prepared under different conditions; (a–l) multilayer gels when (a–d) none of the layers is annealed, (e–h) one layer is annealed and (i–l) both layers are annealed, and (m–o) single layer gel of (1ThNapFF+FmocFF) annealed. (a, e, i, m) In each case, after 16 h, the system was cut into six sections (L1 to L6). (b, f, j, n) Percentage of FmocFF (blue) and 1ThNapFF (red) in the layers L1–L6. (c, g, k, o) Stiffness at γ=0.05 % of the sections L1–L6 obtained from strain sweeps compared to the stiffness of the hydrogel of (c, g, k) FmocFF (blue) or 1ThNapFF (red) obtained before (N/A) and after annealing (A), and (o) (1ThNapFF+FmocFF) (violet) obtained after annealing (A). (d, h, l) Confocal microscopy images of the sections L1–L6 (scale bar is 20 μm).

To demonstrate how annealing can change composition, a layered gel was again formed, with the bottom layer formed from only 1ThNapFF and the top layer from FmocFF (Figure 4e–h, Figure S23–S24). In this case, urea, urease and methyl formate were included only in the 1ThNapFF gel. After 16 hours, the gel was cut into sections. In each section, both the compounds are present and the concentration of 1ThNapFF in the FmocFF layer increases going from the top to the interface as well as the concentration of FmocFF in the 1ThNapFF layer increases going from the bottom to the junction between the two layers (Figure 4f). Confocal microscopy (Figure 4h) shows that the top layers contain spherulites very similar to those from the FmocFF system without annealing as expected. Layer 3 (the closest to the annealed 1ThNapFF) shows the presence of larger spherulites showing that there have been some changes due to the proximity to where the annealing occurred. The bottom layers show a very different microstructure in agreement with these layers have been annealed where significantly larger domains can be seen that are no longer spherulitic in nature. This type of microstructure is typical of our pH triggered gels and similar to what happens when 1ThNapFF alone undergoes such an annealing approach.^[19]^ The top layers show an improvement in the stiffness compared to the previous experiment, suggesting a rearrangement of the fibrous structure, while there are no significant changes in the behavior of the 1ThNapFF layer.

Next, a bilayer gel with both layers undergoing annealing was formed (Figure 4i–l, Figure S25–S26). The bottom layer was formed with only 1ThNapFF and the top layer with only FmocFF and urea, urease and methyl formate were included in both layers. After 16 hours, the gel was cut into sections. Apart from the section L1, both the compounds are present in each section and the mixing is more prominent at the interface (Figure 4j). The layers stiffness has a chiastic trend, as at the interface the layers appear stiffer compared to the edges (Figure 4k). NMR and rheology data are summarized with confocal images of these sections in Figure 4i–l. Confocal microscopy shows the lack of spherulitic domains as expected for pH triggered gels.^[19]^ Long fibers are present in all the gel matrix but are more evident going from top to bottom. It is very clear that the microstructure is heavily affected by annealing.

Finally, as a comparison, a bulk multicomponent gel was prepared with both components mixed throughout containing urea, urease and methyl formate and allowed to stand for 16 hours (Figure 4m–o and Figures S27 and S28). After annealing, the chemical composition throughout the gel matrix is not steady; there is a slight increase in the relative concentration of FmocFF going from top to bottom. Rheologically, there is an improvement in the gel stiffness that follows the same trend (Figure 4o). This observation supports the self‐sorting of the fibers. FmocFF reassembles first and simply driven by gravity moves to the bottom, while 1ThNapFF remains in solution and reassembles later.

In conclusion, we have shown how we can use this annealing approach to convert co‐assembled systems to self‐sorted systems. Initial addition of water to a solution of two gelators in DMSO favours co‐assembly. Such a solvent‐switch gelation is a phase separation process and there seems to insufficient time or driving force for anything other than co‐assembly to occur. Annealing by a pH increase and decrease results in self‐sorted gels. Gelation on a slow pH decrease drives self‐sorting on the basis of the apparent p*K*_a_ of each gelator. Annealing specific layers in two component gels allows complex hierarchical systems to be formed. There is a significant potential here to form many varied systems by changing the composition and rates of pH change allowing access to a far greater range of morphologies and complexity than can be achieved in single component systems.

## Experimental Section

Full experimental details, further rheology, time sweep data, confocal microscopy. The Supporting Information is available free of charge on the Wiley website as a PDF document.

## Conflict of interest

The authors declare no conflict of interest.

## Supporting information

As a service to our authors and readers, this journal provides supporting information supplied by the authors. Such materials are peer reviewed and may be re‐organized for online delivery, but are not copy‐edited or typeset. Technical support issues arising from supporting information (other than missing files) should be addressed to the authors.

Supporting Information

## Data Availability

The data that support the findings of this study are available in the supplementary material of this article.
