# Supplementary material for: Controlled Annealing in Adaptive Multicomponent Gels
Source: Angew Chem Int Ed Engl. 2022 Dec 14;62(4):e202215813. doi: 10.1002/anie.202215813 (PMC10107119; doi:10.1002/anie.202215813)
Supplement: Supplementary file 1 — Supporting Information [file ANIE-62-0-s001.pdf]

Angewandte Chemie
Eine Zeitschrift der Gesellschaft Deutscher Chemiker

Supporting Information

Controlled Annealing in Adaptive Multicomponent Gels

*P. Ravarino, S. Panja, S. Bianco, T. Koev, M. Wallace, D. J. Adams**

Table of Contents

Experimental Details	S2
Supplementary Figures	S9
References	S24

Experimental details

Materials: Compounds 1ThNapFF[1] and FmocFF[2] were synthesised as described previously. Full characterisation data for all precursors and the final FmocFF are provided starting on page S6. Data for 1ThNapFF were provided elsewhere.[1] Urease (U4002-100KU, Jack Beans, 100000 units/g solid) and urea (ultrapure 99%) were purchased from Alfa Aesar. Methyl formate was purchased from Sigma Aldrich. All other chemicals and solvents were obtained from commercial suppliers and used as received. Deionised water was used throughout all experiments.

Preparation of solutions: Stock solutions of 1ThNapFF and FmocFF were prepared in DMSO at a concentration of 20 mg/mL by stirring. The stock solutions of urease were prepared at the concentrations of 0.253 mg/mL and 0.506 mg/mL in H_2O. The enzyme concentration in the stock solution was determined from the mass (in mg) dissolved in known volume of H_2O. Stock solution of the urea was prepared in H_2O in the concentration of 2 M. The enzyme and urea are highly soluble in water at room temperature, and therefore did not require stirring. Solutions of gelator, urease and urea were prepared freshly before each experiment. Stock solution of NaOH was prepared in water at a concentration of 0.1 M.

Hydrogel Preparation: For the single component gels of 1ThNapFF and FmocFF, the hydrogels were firstly prepared in absence and presence of urease. For these gels, either 1.6 mL of H_2O or a mixture of 1.580 mL of urease (0.253 mg/mL), and 0.020 mL of water was added to the mixture of 0.2 mL of DMSO and 0.2 mL of respective solution of the gelator (final concentration 2 mg/mL). The samples were left overnight prior to any measurements. To prepare the multicomponent gels of (1ThNapFF + FmocFF) in DMSO-water, a mixture of 1.580 mL of urease (0.506 mg/mL) and 0.020 mL of water was transferred to the vial containing a mixture of 1ThNapFF and FmocFF present in a volume of 0.2 mL each. To anneal the gels involving the enzymatic reaction, a common procedure was followed. In all cases, urea (either 10 μL or 20 μL depending on whether a single or multicomponent system is being used) and methyl formate (either 100 μL or 150 μL depending on whether a single or multicomponent system is being used) were incorporated into the systems before addition of urease (either 0.253 mg/mL or 0.506 mg/mL) keeping the final ratio of DMSO and H_2O at 20:80 (v/v). Therefore, in all cases, the concentrations of 1ThNapFF and FmocFF were 2 mg/mL. Initial concentration of urease was either 0.01 M or 0.02 M and the initial concentration of urease was either 0.2 mg/mL or 0.4 mg/mL. All these samples were prepared in 7 mL Sterilin vials.
To anneal the single component gels of 1ThNapFF and FmocFF (concentration is 2 mg/mL) a similar methodology was followed. In this case the annealing conditions were used as: [urea] = 0.01 M, [urease] = 0.2 mg/mL, volume of methyl formate added 100 μL. In this case gels were prepared in about 2 mL volume (volume of the solvent) in 7 mL Sterilin vial.

Preparation of multilayer gels: For the preparation of these gels the tip of a 12 mL polypropylene syringe was cut off. The plunger of the syringe was then adjusted to leave about 5 mL of volume and secured on the bench using some blu tack. For each multilayer gel, the bottom layer was prepared directly in the cut syringe, mixing the solution of 1ThNapFF with water in presence and in absence of urea, urease and methyl formate. For these gels, either 1.6 mL of H_2O or a mixture of 1.580 mL of urease (0.253 mg/mL), 0.010 mL of 2M urea in H_2O and 0.010 mL of water was added to the mixture of 0.2 mL of DMSO and 0.2 mL of 1ThNapFF (final concentration 2 mg/mL). After about 45 seconds from the addition of H_2O, the second gel layer was prepared onto the first one. In this case of 0.2 mL of DMSO, 0.2 mL of the FmocFF solution and 1.6 mL of H_2O (final concentration 2 mg/mL) were mixed in a 7 mL sterilin vial and gently poured onto the first layer before gelation occurred. Four reference samples consisting of single-layer gels of the single components were prepared directily in the cut syringe. For these gels, either 1.6 mL of H_2O or a mixture of 1.580 mL of urease (0.253 mg/mL), 0.010 mL of 2M urea in H_2O and 0.010 mL of water was added to the mixture of 0.2 mL of DMSO and 0.2 mL of the respective solution of the gelator (final concentration 2 mg/mL). Finally, a bulk multicomponent gel of about 4.2 mL of volume was prepared mixing 0.4 mL of 1ThNapFF solution, 0.4 mL of FmocFF solution, 0.040 mL of 2 M urea in H_2O, 3.16 mL of urease (0.506 mg/mL) and 0.2 mL of methyl formate. Each of these gels were covered with some parafilm and left to rest for 16 hours, then they were extruded

with the plunger and cut into equal parts to obtain either 6 (in the case of the bilayer and the bulk multicomponent gels) or 3 (in the case of the reference samples) sections.

pH measurements: A FC200 pH probe from HANNA instruments with a 6 mm x 10 mm conical tip was used for pH measurements. The stated accuracy of the pH measurements is ±0.1. For the urea-urease reaction involving the gelator, the reaction mixtures were prepared as described above at a 2 mL volume in a 7 mL Sterilin vial and the pH change was monitored with time. The temperature was maintained at 25 °C during the measurement by using a circulating water bath.
pK_a determination for the individual components was carried out by recording the pH values after each addition of HCl (0.1M) to the solution of the respective gelator (concentration of is 2 mg/mL) containing 1 molar equivalents of NaOH (0.1 M) in 20% DMSO in H_2O. For the multicomponent system (1ThNapFF + FmocFF), a similar procedure was followed. In this case, HCl was gradually added to the mixture of (1ThNapFF + FmocFF) (concentration of the individual components is 2 mg/mL) containing 0.192 mL of NaOH (0.1 M) in 20% DMSO in H_2O. In all cases, to prevent any gel formation during the titration, the solutions were stirred continuously.

Rheological measurements: All rheological measurements were undertaken on an Anton Paar Physica MCR 101 rheometer at 25 °C. For gels prepared in sterilin cups, strain, frequency, and time sweeps were performed using a vane and cup geometry. Strain sweeps were performed at 10 rad/s from 0.01 % to 1000 % strain. Frequency sweeps were carried out from 1 rad/s to 100 rad/s at 0.5 % strain. Time sweeps were performed at an angular frequency of 50 rad/s and with a strain of 0.5%. For these experiments, gels were prepared as mentioned earlier in 2 mL volume in a 7 mL Sterilin vials. For gels sections of single- and double layer systems measurements a parallal plate geometry (d = 12.5 mm) was used for strain sweep experiments. The gel disks were transferred onto a piece of sandpaper secured on the rheometer plate with some tape. Additional strain sweep experiments were carried out on gels sections of single-layer gels of the single components to compare the results obtained for the bilayer system with the same geometry. Strain sweeps were performed at 10 rad/s from 0.01 % to 1000 % strain. For these experiments, gels were prepared as mentioned earlier in a 12 mL cut syringe. All gels were left ~16 hours before being measured.
Long-term time sweeps were performed on an Anton Paar Physica MCR 301 rheometer at 25 °C. The time sweeps were collected using an angular frequency of 50 rad/s and strain of 0.5%. For these measurements, the samples were prepared immediately before positioning the vial in the rheometer cup system. The data was collected over a period of 64 hours. To prevent evaporation of the gel over time, aluminium foil was placed around the measuring system.

Circular dichroism: Data were collected using a Chirascan VX spectrometer (applied photophysics) using a 0.01 mm path length quartz cuvette. All spectra were aquired at 25 °C with a scanning step size of 1.0 nm, scanning rate of 0.25 s, in the range 180-400 nm. All gel samples were prepared in 2 mL volume in Sterilin vials using the same methodology as described earlier and were left overnigh. Then, small amounts of the gels were deposited on the quartz cuvette (path length is 0.01 mm) for measurement. For these experiments, concentration of 1ThNapFF and FmocFF is 2 mg/mL, the concentration of urease is 0.2 mg/mL (FmocFF alone and 1ThNapFF alone) and 0.4 mg/mL (multicomponent system). Initial concentration of urea is 0.02 M and volume of methyl formate added is 100 μL.

NMR spectroscopy experiments: ^1H NMR spectra (Figures S1-S4, S19, S21, S23, S25, S27) were recorded on a Bruker Avance III 400 MHz instrument. All the gels were initially prepared following the same methodology described in the section "Preparation of multilayer gels", dividing each section and freeze-drying them separately to remove the solvent. To prepare the samples for NMR spectroscopy, the freeze-dried samples were dissolved in 0.5 mL of d_6-DMSO.

^1H NMR spectra to follow the annealing process (Figure 3c and 3d, S9-S14) were recorded at 298 K on a Bruker 500 MHz Avance III spectrometer with 16 scans and two dummy scans, a 30° pulse, relaxation delay of 1 s and signal acquisition time of 3.2 s. Spectra were referenced to the residual ^1H signal from the d_6-DMSO at 2.60 ppm. The sample in the presence of urea-urease (Figure 3c, main text) was prepared directlty in a 5 mm diameter NMR tube in D_2O and

d_6-DMSO following the section above (hydrogel preparation) but with the volumes adjusted to achieve a total volume of 550 μL. Immediately upon addition of the D_2O, the sample was shaken vigorously while still turbid and the tube immediately centrifuged (< 1000 rpm) for 5 s on a Hettich H1011 hand centrifuge to drive away the air bubbles. This shaking and centrifugation step was found to be necessary to achieve the homogenous mixing in the 5 mm tube required to obtain good quality NMR spectra. The single component 1ThNapFF sample sample in the presence of urea-urease and methyl formate (Figure S25) was similarly prepared, following the relative quantities of reagents described above. The two component sample that was raised from low to high pH using urea-urease, without methyl formate (Figure 3d, main text) was prepared as for the sample with methyl formate, but with only 10 μg/mL of urease and 10 mM ammonium formate to act as a reference for ^1H integration.

Confocal microscopy: A Zeiss LSM710 confocal microscope (Zeiss, Göttingen, Germany) with an LDEC Epiplan NEUFLUAR 50X, 0.55 DIC (Carl Zeiss, White Plains, NY, USA) objective was used for imaging. All gel samples were prepared in presence of Nile blue (2 μL/mL of a 0.1 wt % solution in water). The gels described in the section "Hydrogel preparation" which did not involve the use of methyl formate, urea and urease were prepared directly in CELLview culture dishes by depositing 40 μL of the gelator solution in DMSO. For 1ThNapFF and FmocFF 20 μL of the corresponding peptide were mixed with 20 μL of DMSO, for (1ThNapFF + FmocFF) 20 μL of each solution were mixed, then 160 μL of H_2O were added onto the DMSO solutions. The culture dishes were covered with their own lid and the samples were left to rest for 16 hours in a closed box with a wet piece of blue roll to prevent drying of the samples. The gels descrbed in the section "Hydrogel preparation" which instead involved the use of urea, urease and methyl formate were prepared following the procedure describe in the same section in a volume of about 2 mL of solvent in 7 mL Sterilin vials. Then a small amount of each section was deposited onto glass microscope slides. A cover slip was gently placed on the gel. The gels sections were prepared as described above in the section "Preparation of multilayer gels" in 12 mL cut syringes and cut after 16 hours. Then a small amount of each section was deposited onto glass microscope slides. A cover slip was gently placed on the gel. All the samples were excited at 633 nm using a He-Ne laser. Images were captured using Carl Zeiss ZEN 2011 v7.0.3.286 software.

Synthesis of FmocFF

tert-Butyl (2*S*)-2-[(2*S*)-2-({[(9*H*-fluoren-9-yl)methoxy]carbonyl}amino)-3-phenylpropanamido]-3-phenylpropanoate (**FG-005**)

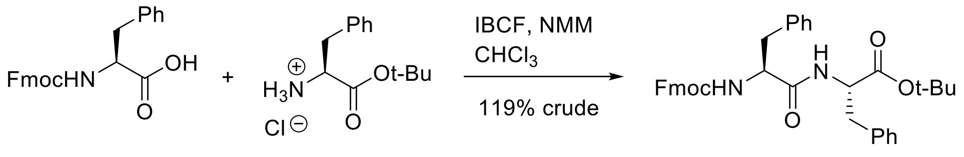

To a solution of Fmoc-phenylalanine (5.62 g, 14.5 mmol) in chloroform (70 mL) was added *N*-methylmorpholine (1 eq, 1.59 mL) followed by *iso*-butyl chloroformate (1 eq, 1.89 mL). After stirring for 5 minutes, phenylalanine *tert*-butyl ester hydrochloride (1.05 eq, 3.92 g) and another portion of *N*-methylmorpholine (1 eq, 1.59 mL) were added and the reaction mixture was stirred overnight. It was then diluted with chloroform, washed in turn with 1M hydrochloric acid, water, and brine, dried ($MgSO_4$), and evaporated under reduced pressure. The crude title compound was thus obtained in 119% (10.2 g) yield as an off-white solid and used as such in the next step. A small amount was purified *via* column chromatography (1:99 ethyl acetate/dichloromethane, $R_f \approx 0.1$) to afford an analytical sample. Proton NMR suggests the presence of rotamers in a *ca.* 8:2 ratio, which also causes the doubling of some of the carbon NMR signals.

δ_H (400 MHz, DMSO-d_6) 8.52 (0.2 H, d, *J* 7.42, N<u>H</u>), 8.42 (0.8 H, d, *J* 7.32, N<u>H</u>), 7.87 (2H, d, *J* 7.59, $\underline{H}_{Ar}$), 7.65-7.60 (2.6 H, m, $\underline{H}_{Ar}$ and N<u>H</u>), 7.46-7.10 (14.4 H, m, $\underline{H}_{Ar}$), 4.47-4.27 (2 H, m, C<u>H</u>*), 4.19-4.00 (3 H, m, O$C\underline{H}_2C\underline{H}$), 3.01-2.93 (3H, m, Ph$C\underline{H}_2$), 2.75 (1H, dd, *J* 13.56, 11.22, Ph$C\underline{H}_2$), 1.31 (9H, s, $C(C\underline{H}_3)_3$). δ_C (100 MHz, DMSO-d_6) 171.70, 170.40, and 155.76 (<u>C</u>=O), 143.77, 143.72, 140.65, 138.14, 137.09, 129.25, 129.22, 128.17, 128.02, 127.62, 127.04, 126.51, 126.25, 125.36, 125.27, and 120.07 ($\underline{C}_{Ar}$), 80.66 ($\underline{C}(CH_3)_3$), 65.66 (O$\underline{C}H_2CH$), 55.86 (<u>C</u>H*), 54.26 (<u>C</u>H*), 46.54 ($OCH_2\underline{C}H$), 37.49 (Ph$\underline{C}H_2$), 36.81 (Ph$\underline{C}H_2$), 27.51 ($C(\underline{C}H_3)_3$). HRMS (ESI) m/z: $[M+Na]^+$ calcd for $C_{37}H_{38}N_2NaO_5$ 613.2673; found 613.2666.

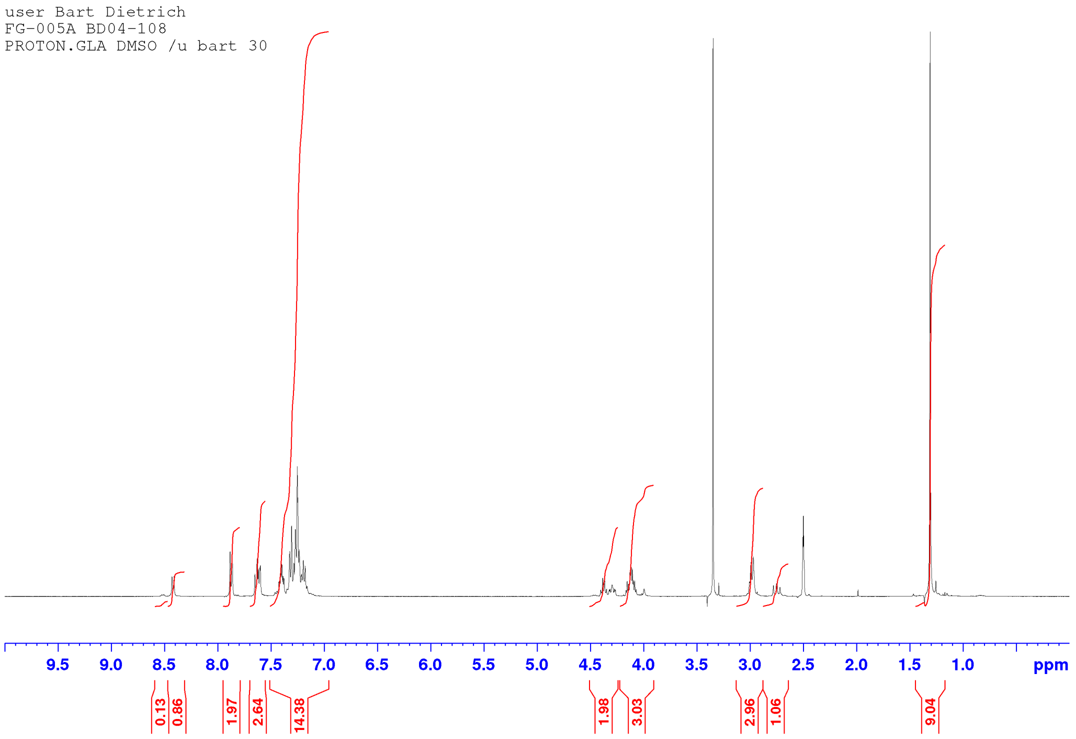

Figure S1. Proton NMR of **FG-005**.

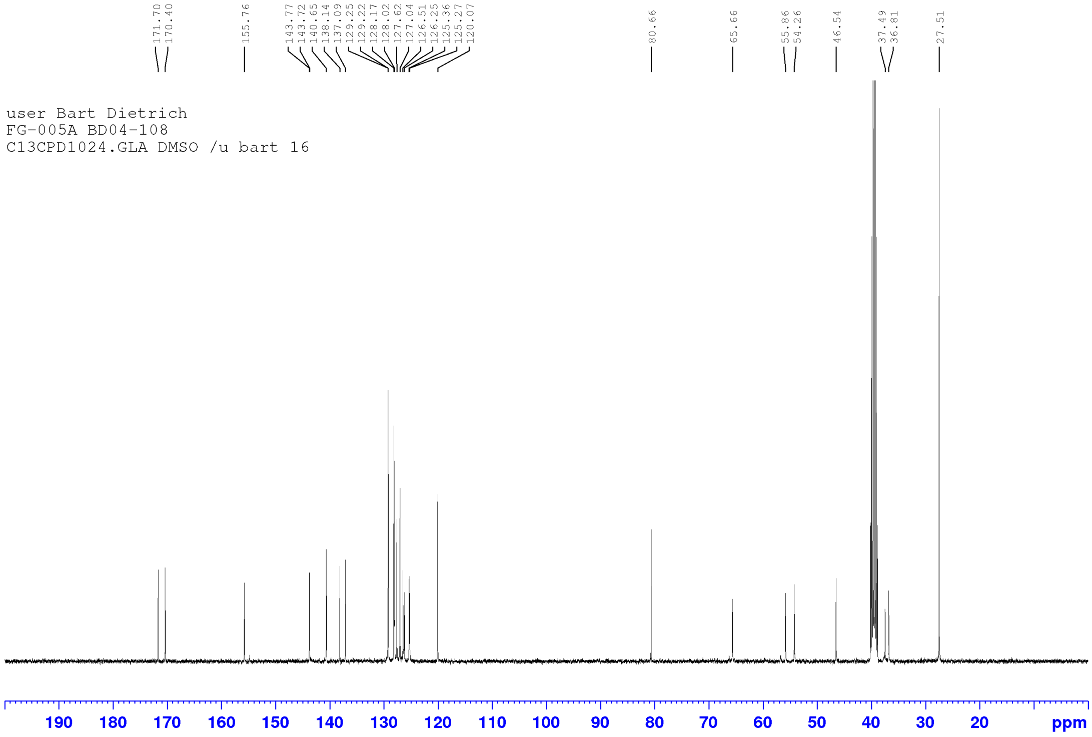

Figure S2. Carbon NMR of **FG-005**.

(2*S*)-2-[(2*S*)-2-({[(9*H*-Fluoren-9-yl)methoxy]carbonyl}amino)-3-phenylpropanamido]-3-phenylpropanoic acid (FmocFF, **FG-007**)

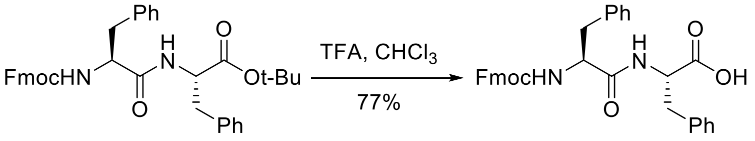

FG-005 **FG-007**

To a solution of crude **FG-005** (8.27 g, 14.0 mmol) in chloroform (40 mL) was added trifluoroacetic acid (*ca.* 18 eq, 20 mL) and the solution was stirred overnight. After this time, diethyl ether (*ca.* 200 mL) was added to the reaction mixture resulting in a white precipitate. This was stirred for 20 minutes, filtered, and washed with diethyl ether in the filter. The solid was transferred into a conical flask, then slurried in diethyl ether and decanted twice. Residual solvents were removed from the solid on a rotary evaporator, then by storing in an oven at 80 °C overnight. The title compound was thus obtained as a white solid in 77% (5.97 g) yield containing *ca.* 0.3% residual diethyl ether. The presence of rotamers (in a *ca.* 1:9 ratio) complicates the proton and carbon NMR spectra causing peak splitting.

δ_H (400 MHz, DMSO-d_6, 25 °C) 12.79 (1H, br s, COOH), 8.42 (0.12 H, d, *J* 7.55, NH), 8.28 (0.88 H, d, *J* 7.77, NH), 7.87 (2H, d, *J* 7.59, H_{Ar}), 7.64-7.09 (17 H, m, NH and H_{Ar}), 4.55-4.52 (0.12 H, m, CH*), 4.50-4.44 (0.88 H, m, CH*), 4.36-4.32 (0.12 H, m, CH*), 4.30-4.24 (0.88 H, m, CH*), 4.18-3.97 (3 H, m, OCH_2CH), 3.08 (1H, dd, *J* 13.89, 5.19, $PhCH_2$), 2.98-2.91 (2H, m, $PhCH_2$), 2.72 (1H, dd, *J* 13.63, 11.07, $PhCH_2$). δ_C (100 MHz, DMSO-d_6, 25 °C) 172.79, 171.65, and 155.73 (C=O), 143.80, 143.73, 140.68, 138.14, 137.37, 129.27, 129.18, 128.21, 128.04, 127.65, 127.08, 126.48, 126.25, 125.37, 125.28, and 120.08 (C_{Ar}), 65.70 (OCH_2CH), 55.70 (CH*), 53.50 (CH*), 46.58 (OCH_2CH), 37.44 ($PhCH_2$), 36.74 ($PhCH_2$). HRMS (ESI) m/z: $[M+Na]^+$ calcd for $C_{33}H_{30}N_2NaO_5$ 557.2047; found 557.2045.

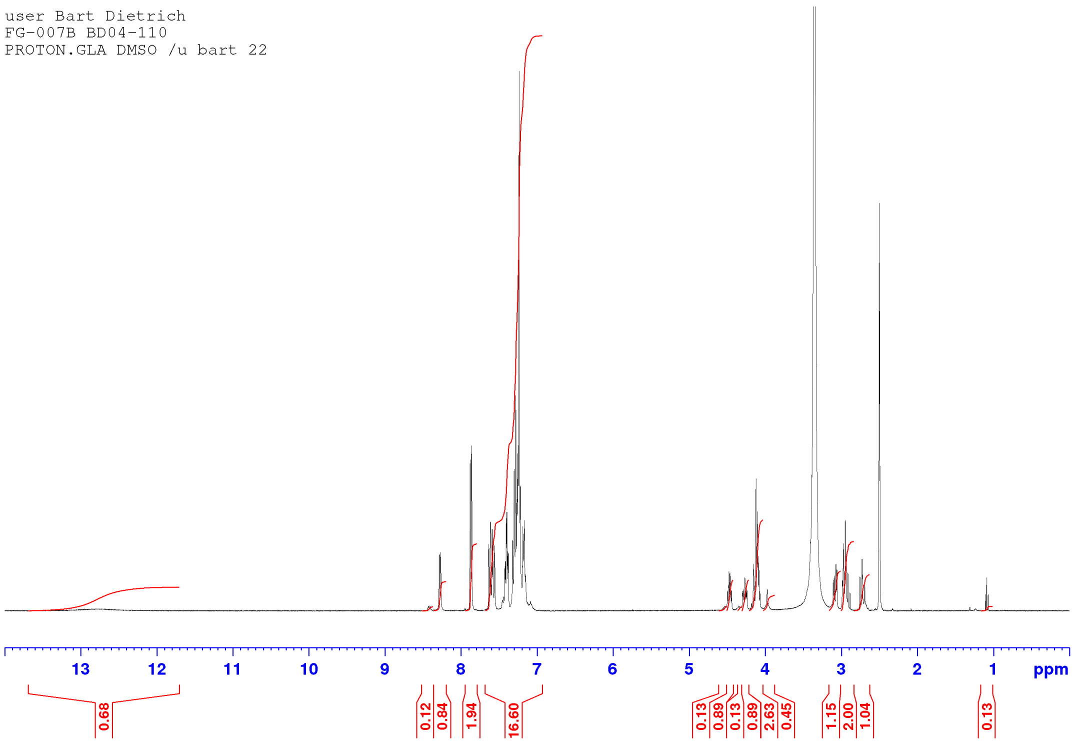

Figure S3 Proton NMR spectrum of **FG-007** (FmocFF)

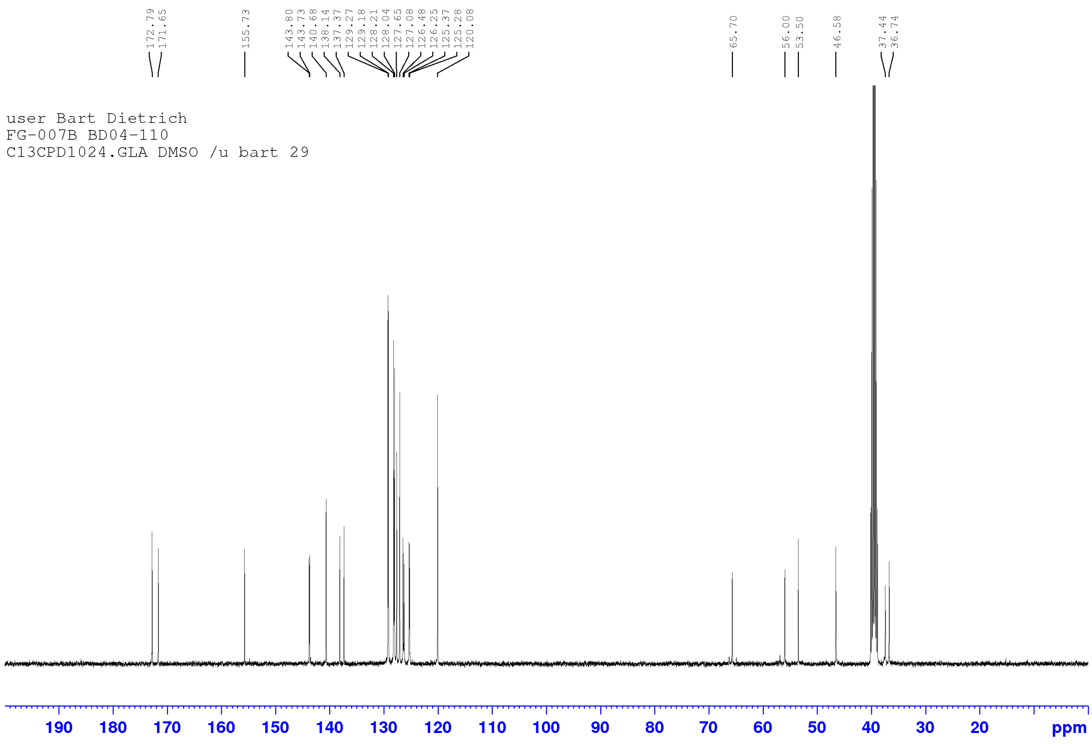

Figure S4. Carbon NMR spectrum of **FG-007** (FmocFF)

Supplementary Figures

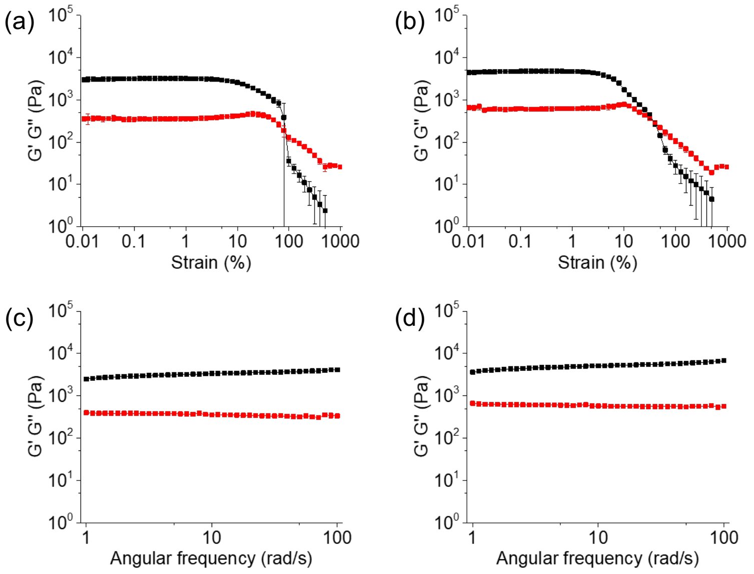

Figure S5. Strain (a, b) and frequency (c, d) sweeps for the hydrogel of 1ThNapFF (a, c) and of FmocFF (b, d). In all cases, concentrations of 1ThNapFF and FmocFF are 2 mg/mL, solvent is DMSO/water (20/80, v/v). The black symbols represent G′, the red symbols G″.

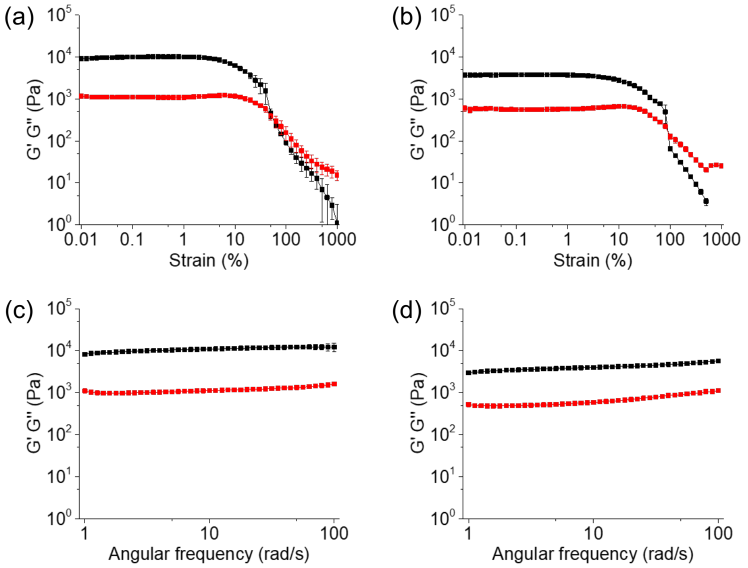

Figure S6. Strain (a, b) and frequency (c, d) sweeps for the hydrogel of (1ThNapFF + FmocFF) in absence (a, c) and presence of 0.4 mg/mL of urease (b, d). In all cases, concentrations of 1ThNapFF and FmocFF are 2 mg/mL, solvent is DMSO/water (20/80, v/v). The black symbols represent G′, the red symbols G″.

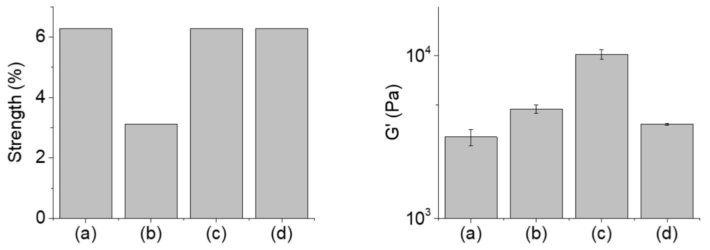

Figure S7. Bar graph demonstrating (left) the gel strength (calculated from strain sweep data, the strain at which the gel stats to break) and (right) gel stiffness (G′ calculated at 0.5% strain from strain sweep) for the hydrogel of (a) 1ThNapFF, (b) FmocFF, (c) (1ThNapFF + FmocFF) and (d) (1ThNapFF + FmocFF) in presence of 0.4 mg/mL of urease. In all cases, concentrations of 1ThNapFF and FmocFF are 2 mg/mL, solvent is DMSO/water (20/80, v/v).

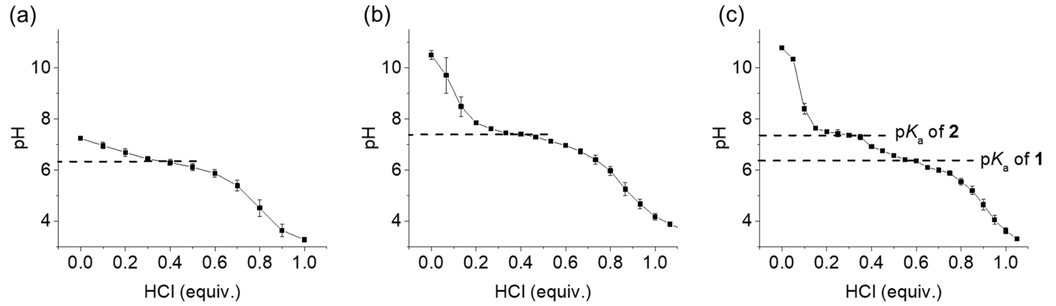

Figure S8. Determination of apparent pK_a of 1ThNapFF (a), FmocFF (b) and (1ThNapFF + FmocFF) (c) in DMSO/H_2O (20/80, v/v). The plateau is taken to represent the apparent pK_a value, shown by the dashed lines. In all cases, concentration of the individual components is 2 mg/mL.

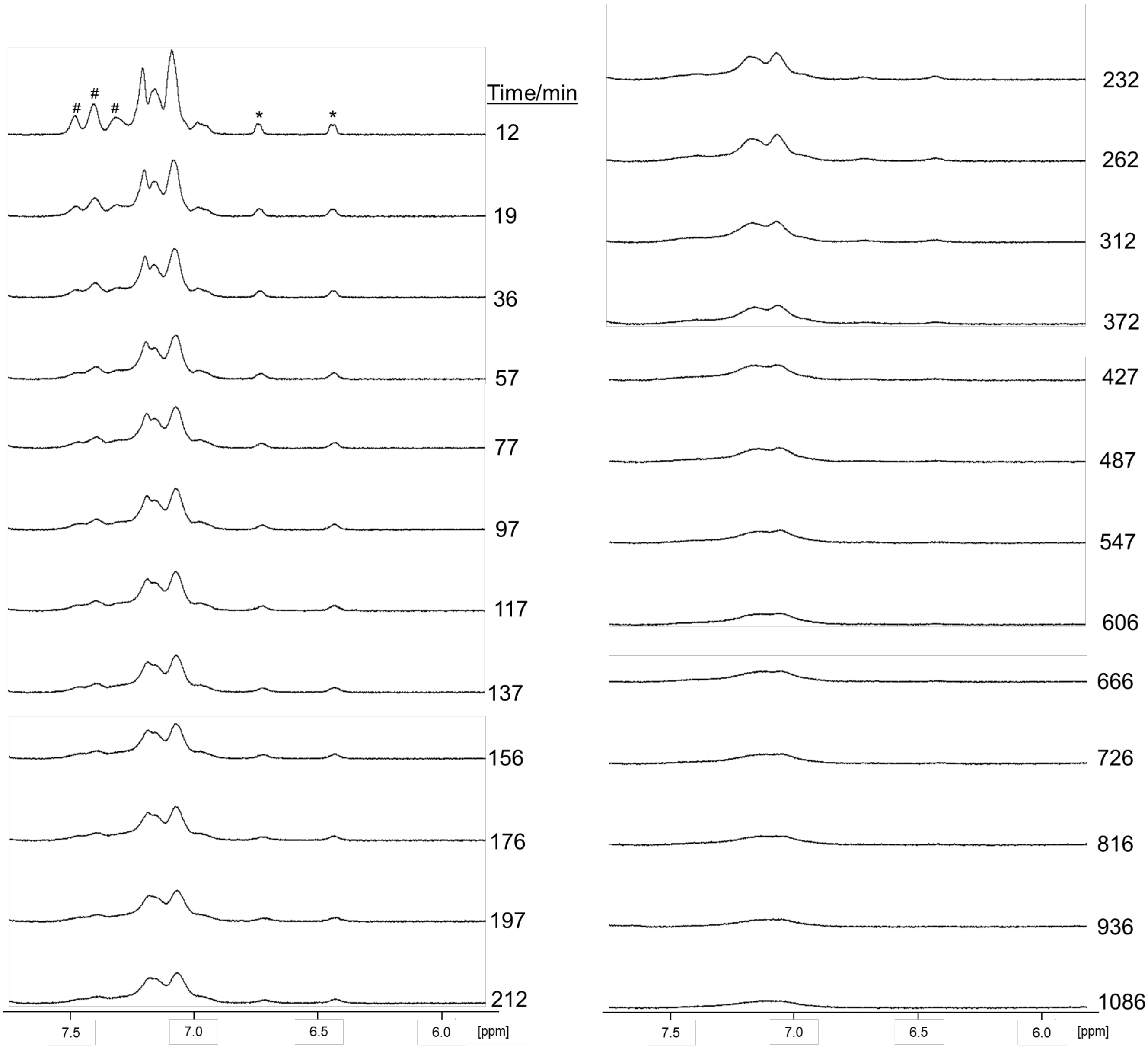

Figure S9. ^{1}H spectra of aromatic region of two component gel during annealing at time indicated since preparation of sample. Peaks marked * belong specifically to 1ThNapFF. Peaks marked # belong specifically to FmocFF. These resonances could not be integrated separately. Spectra were processed with an exponential line broadening factor of 0.3 Hz with manual baseline adjustment to remove the influence of the methyl formate resonance to allow integration of the resonances over time (see Figure 3d).

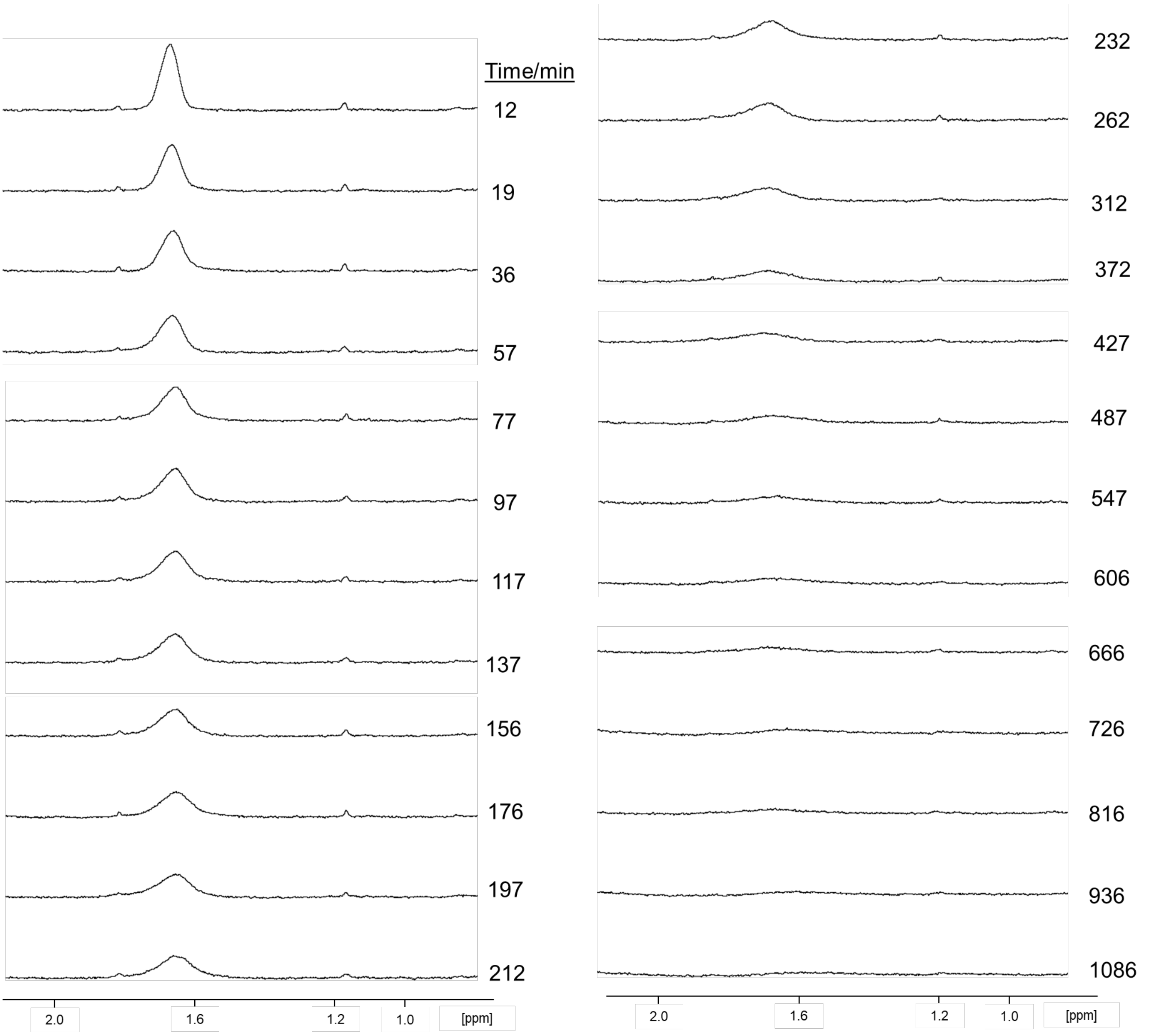

Figure S10. ^{1}H spectra of two component gel during annealing, showing singlet resonance of protons at 6 and 7 position of tetrahydrohydronapthol ring at time indicated since preparation of the sample. Spectra were processed with an exponential line broadening factor of 1 Hz. Integrals of this resonance are presented on Figure 3d.

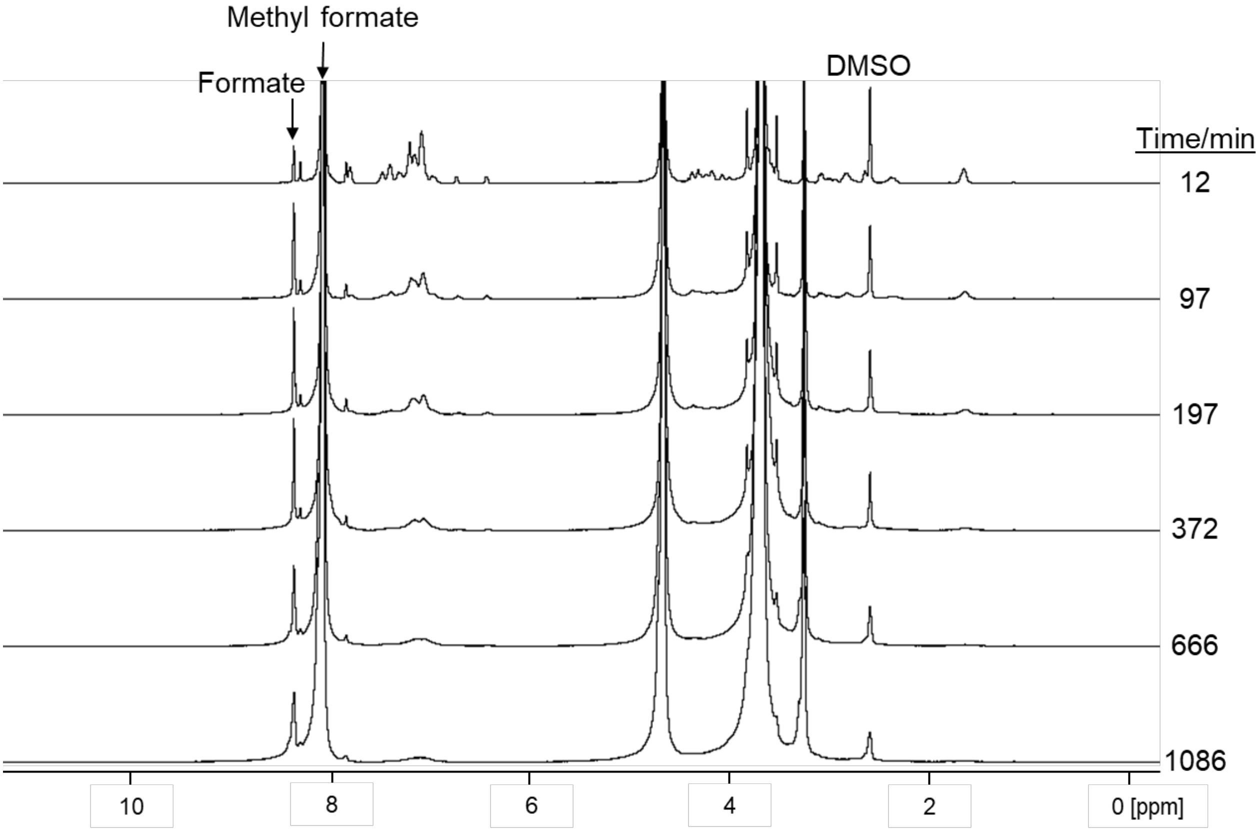

Figure S11. Full ^{1}H spectra of two of two component gel of Figure 3d during annealing at time indicated since preparation of the sample. Spectra were processed with an exponential line broadening factor of 1 Hz.

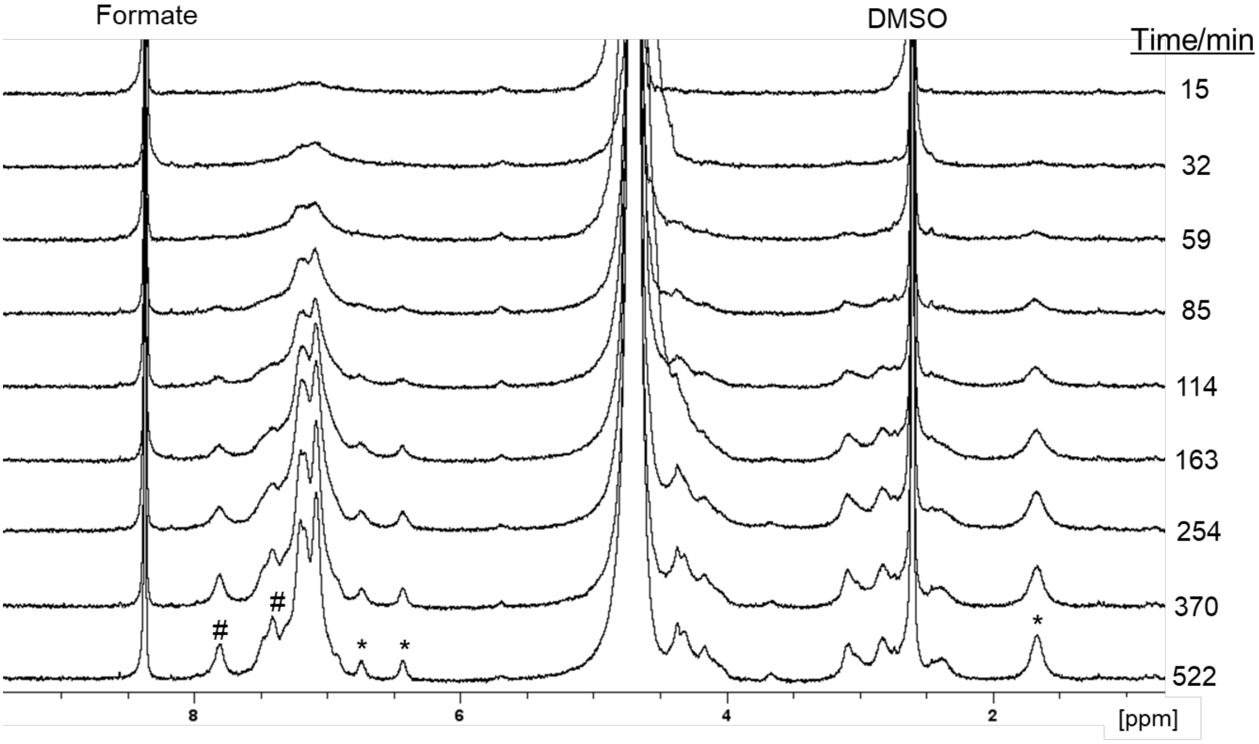

Figure S12. Example ^{1}H spectra recorded at times indicated since preparation of two component sample as pH was raised with urea-urease in absence of methyl formate (Figure 3c). Spectra were processed with an exponential line broadening factor of 1 Hz. Peaks marked * belong specifically to 1ThNapFF. Peaks marked # belong specifically to FmocFF.

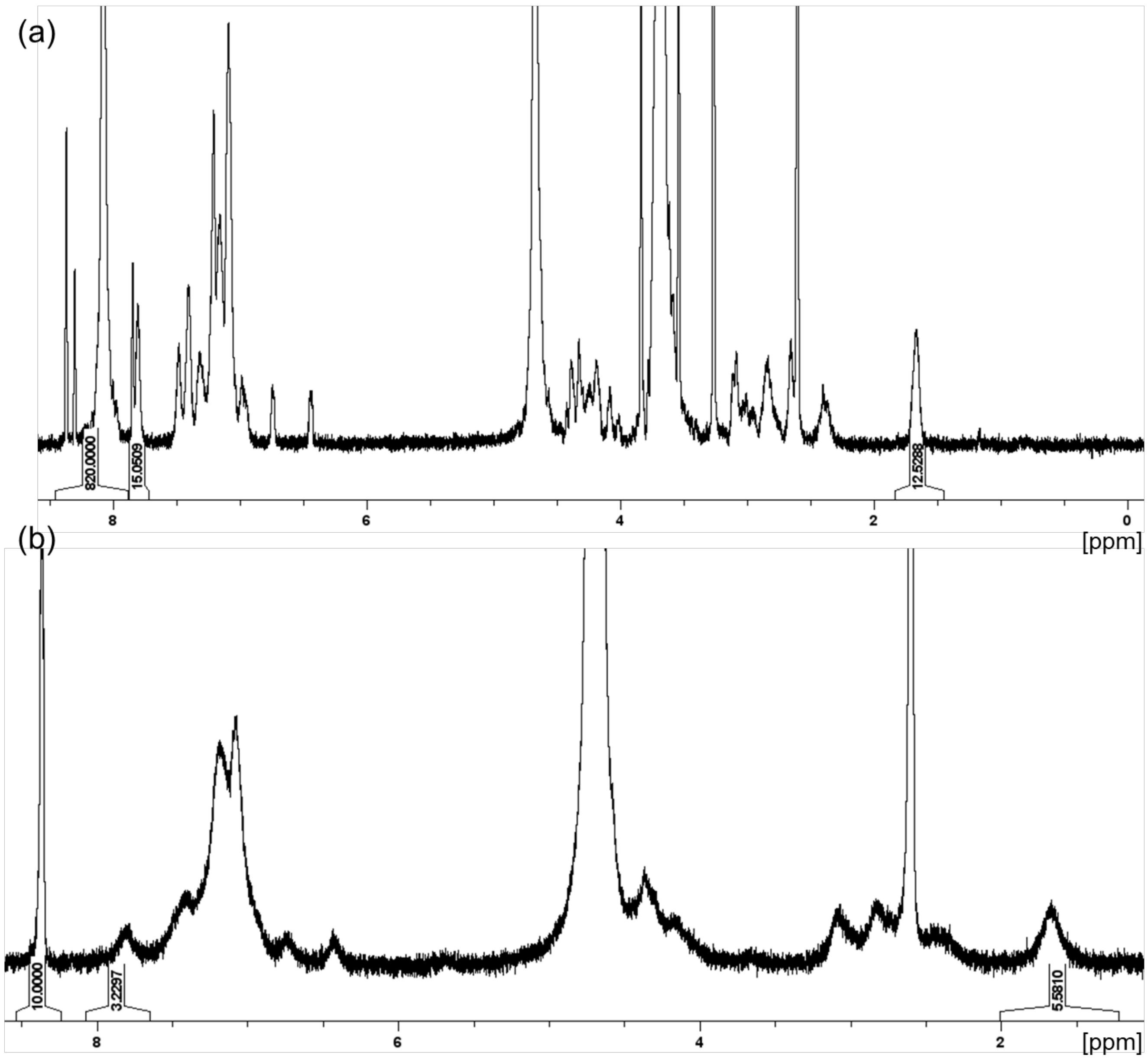

Figure S13. ^{1}H spectra of two component gel prepared with urea-urease and methyl formate (Figure 3d), 12 minutes after preparation (a) and two component gel prepared without methyl formate (Figure 3c), 254 minutes after preparation (b). Spectra were processed without exponential line broadening. Integrals are indicated, calibrated relative to formate. In (a), concentration of formate is 820 mM, indicating that an apparent 3.1 mM of 1ThNapFF and 5 mM FmocFF are NMR visible. We note that these integrals are affected by the short relaxation delay required to capture this initial time point and are thus not strictly quantitative. Theoretical concentrations are 3.7 mM for FmocFF and 4.0 mM 1ThNapFF for 2 mg/mL of both gelators. In (b), formate concentration is 10 mM, indicating that 1.6 mM of FmocFF and 2.8 mM of 1ThNapFF are visible by NMR.

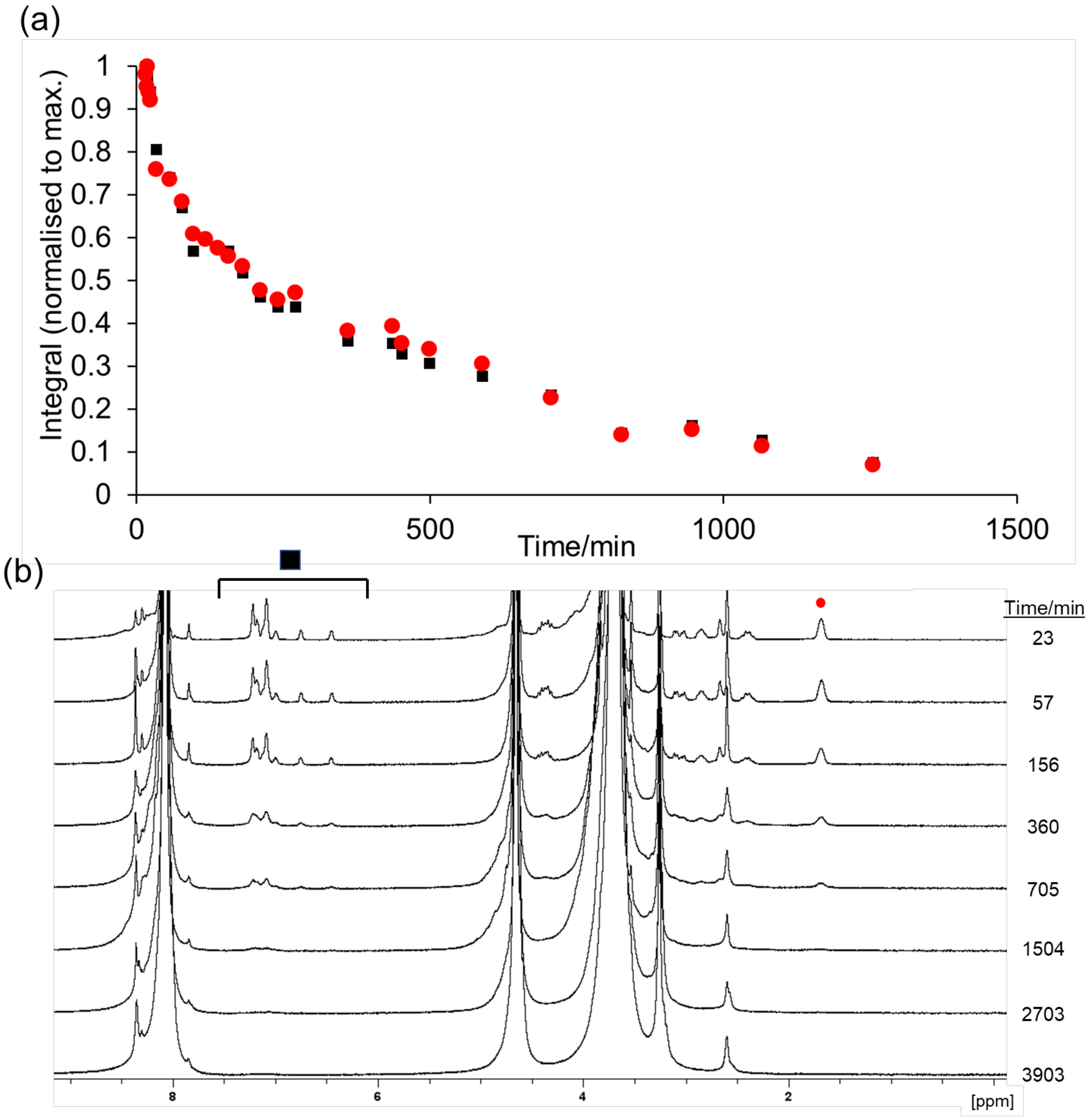

Figure S14. (a) Plot of ^{1}H integral versus time of single component 1ThNapFF sample in the presence of urea-urease and methyl formate. Aromatic resonances (7.6-6.2 ppm, black square) and singlet resonance of protons at 6 and 7 position of tetrahydrohydronapthol ring (red circle). Integrals are plotted as absolute integrals, normalised relative to the largest integral recorded in the data series. (b) Example ^{1}H spectra recorded at times indicated since preparation of the sample. The integral regions plotted on (a) are indicated. Spectra were processed with an exponential line broadening factor of 0.3 Hz.

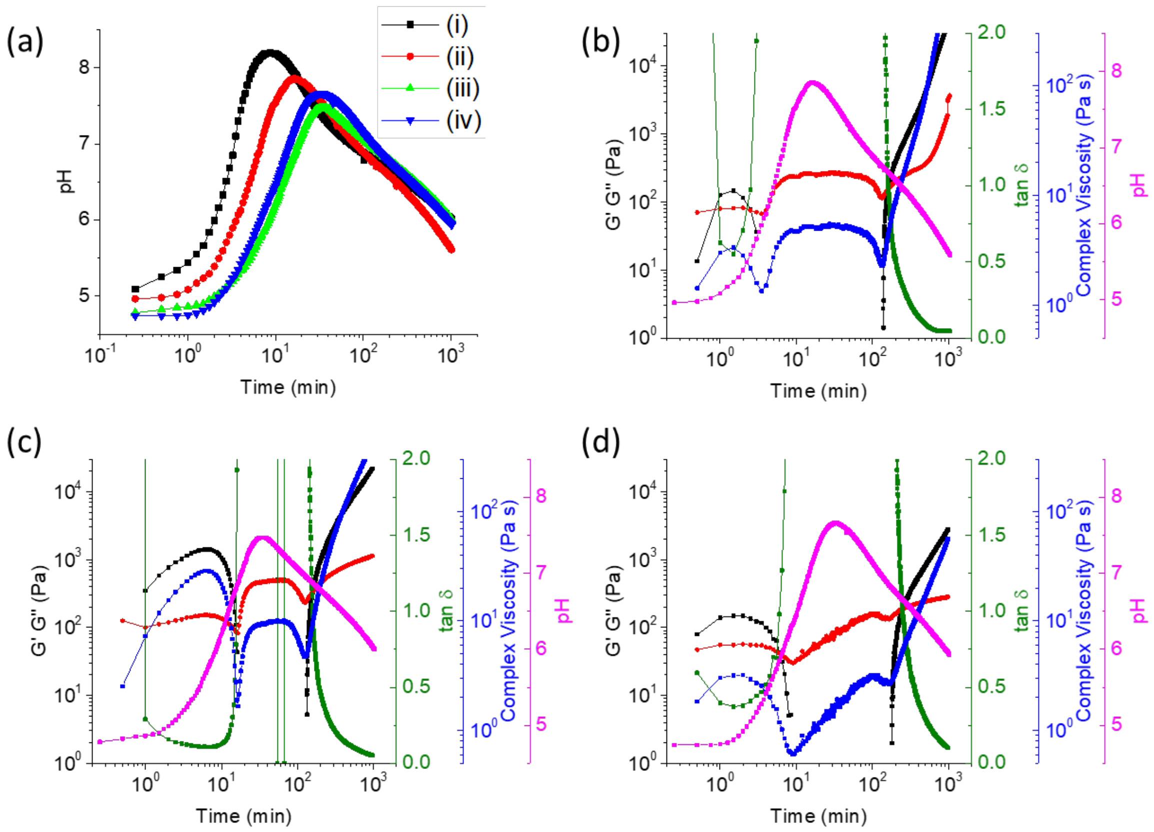

Figure S15. (a) Variation of pH for the multicomponent gel (1ThNapFF + FmocFF) in presence of urea-urease reaction under different conditions: (i) [urease] = 0.4 mg/mL, [urea] = 0.02 M, volume of methyl formate = 100 μL in black; (ii) [urease] = 0.4 mg/mL, [urea] = 0.02 M, volume of methyl formate = 150 μL in red; (iii) [urease] = 0.4 mg/mL, [urea] = 0.01 M, volume of methyl formate = 100 μL in green; (iv) [urease] = 0.2 mg/mL, [urea] = 0.02 M, volume of methyl formate = 100 μL in blue. (b-d) Variation of pH (magenta), G' (black), G" (red), tanδ (green) and complex viscosity (blue) with time for the multicomponent gel (1ThNapFF + FmocFF) involving the conditions (ii)-(iv) mentioned in Figure (a). In all cases the concentration of 1ThNapFF and FmocFF is 2 mg/mL and the solvent is DMSO/H_2O (20:80 v/v).

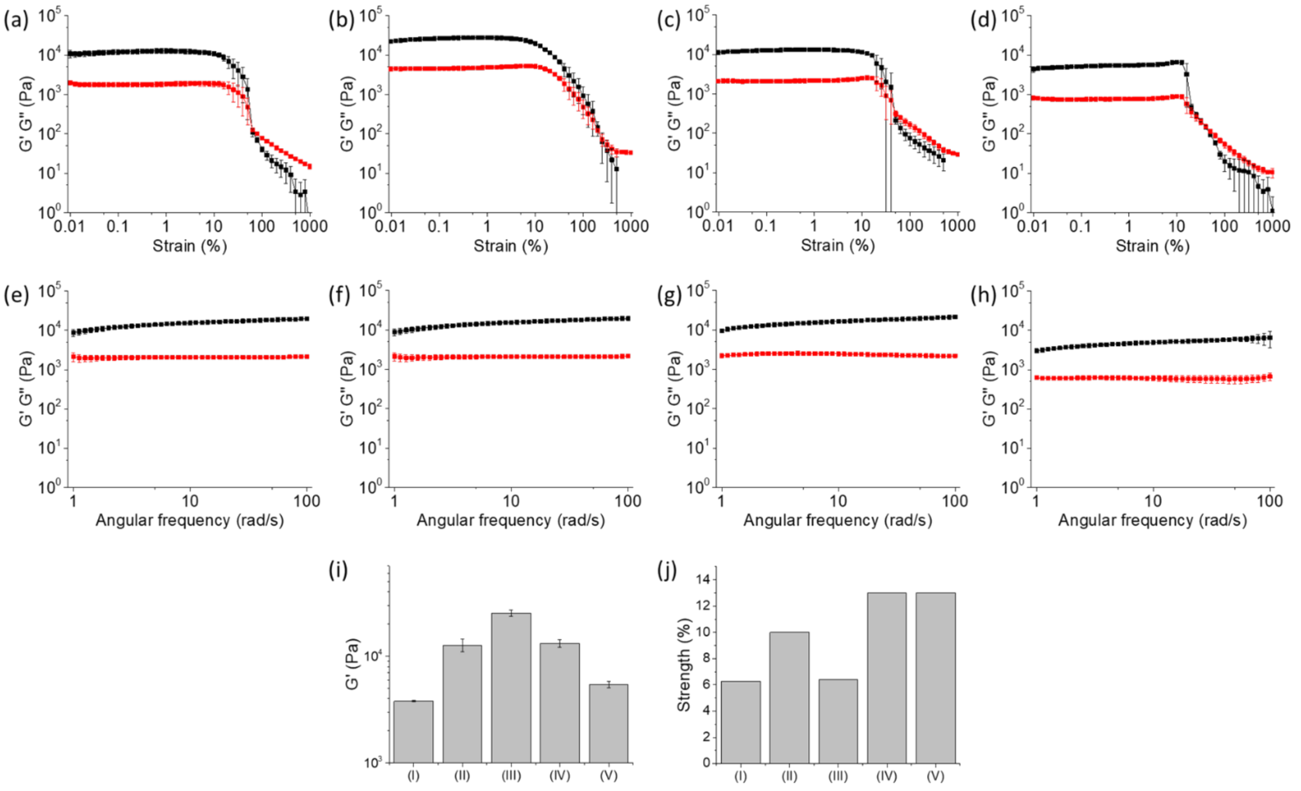

Figure S16. Strain (a-d) and frequency (e-h) sweeps for the multicomponent gel (1ThNapFF + FmocFF) obtained after annealing involving the initial concentrations: (a, e) [urease] = 0.4 mg/mL, [urea] = 0.02 M, volume of methyl formate = 100 μL; (b, f) [urease] = 0.4 mg/mL, [urea] = 0.02 M, volume of methyl formate = 150 μL; (c, g) [urease] = 0.4 mg/mL, [urea] = 0.01 M, volume of methyl formate = 100 μL; (d, h) [urease] = 0.2 mg/mL, [urea] = 0.02 M, volume of methyl formate = 100 μL. In all cases the concentration of 1ThNapFF and FmocFF is 2 mg/mL and the solvent is DMSO/H_2O (20:80 v/v). (i, j) Bar graph representing the changes in (i) stiffness (G') and (j) gel strength of the multicomponent gel (I) after annealing (II – V) involving the conditions used for (a-d) respectively. In all cases, the data were collected after 1000 minutes; the gels are still evolving as can be seen in Figure S8, but we use this time point to exemplify the differences between the systems.

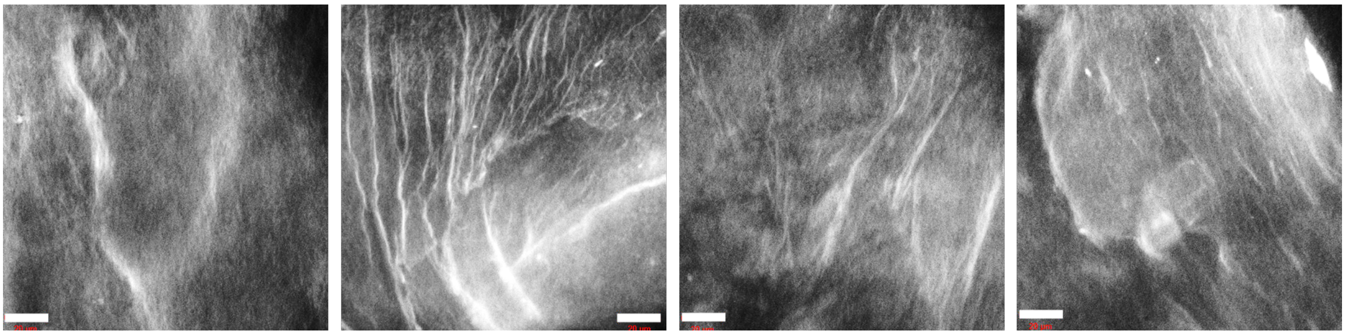

Figure S17. Confocal microscope images of the gels of (1ThNapFF + FmocFF) after annealing involving different initial concentrations. From left to right: (i) [urease] = 0.4 mg/mL, [urea] = 0.02 M, volume of methyl formate = 100 μL; (ii) [urease] = 0.4 mg/mL, [urea] = 0.02 M, volume of methyl formate = 150 μL; (iii) [urease] = 0.4 mg/mL, [urea] = 0.01 M, volume of methyl formate = 100 μL; (iv) [urease] = 0.2 mg/mL, [urea] = 0.02 M, volume of methyl formate = 100 μL. In all cases the concentration of 1ThNapFF and FmocFF is 2 mg/mL and the solvent is DMSO/H_2O (20:80 v/v). Scalebar is 20 μm. In all cases, the data were collected after 1000 minutes; the gels are still evolving as can be seen in Figure S7, but we use this time point to exmplify the differences between the systems.

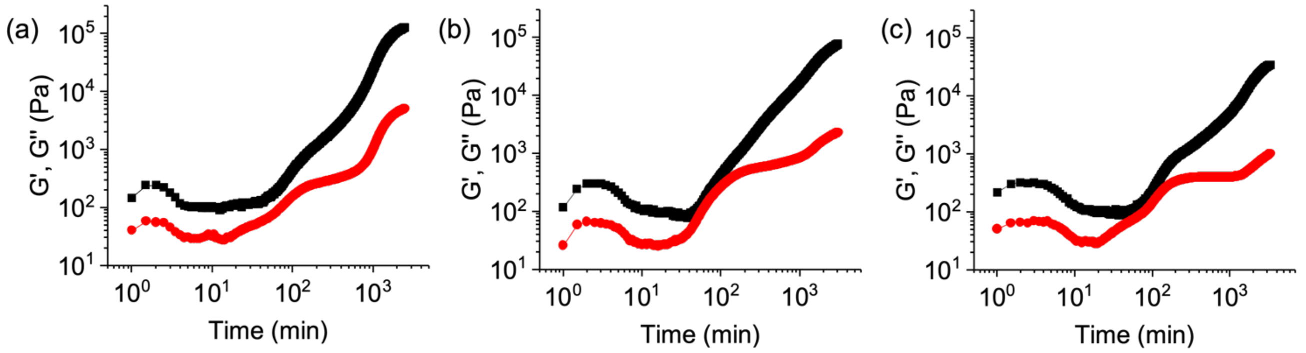

Figure S18. Variation of G' (black) and G" (red) over an extended period (2 days). (a) [urease] = 0.4 mg/mL, [urea] = 0.02 M, volume of methyl formate = 150 μL in red; (b) [urease] = 0.4 mg/mL, [urea] = 0.01 M, volume of methyl formate = 100 μL in green; (c) [urease] = 0.2 mg/mL, [urea] = 0.02 M, volume of methyl formate = 100 μL. In all cases the concentration of 1ThNapFF and FmocFF is 2 mg/mL and the solvent is DMSO/H_2O (20:80 v/v).

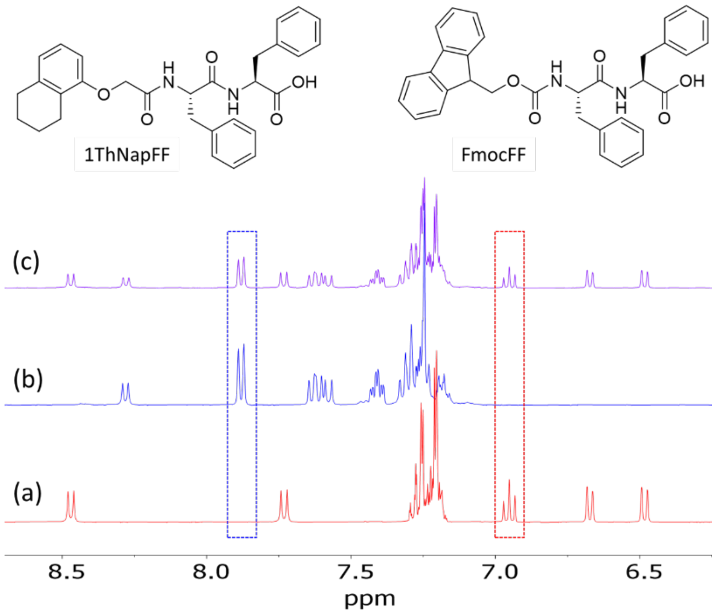

Figure S19. Partial proton NMR spectra of (a) 1ThNapFF, (b) FmocFF and (c) (1ThNapFF + FmocFF) in d_6-DMSO. The red and blue rectangles represent characteristic proton signals for 1ThNapFF and FmocFF in the multicomponent system respectively.

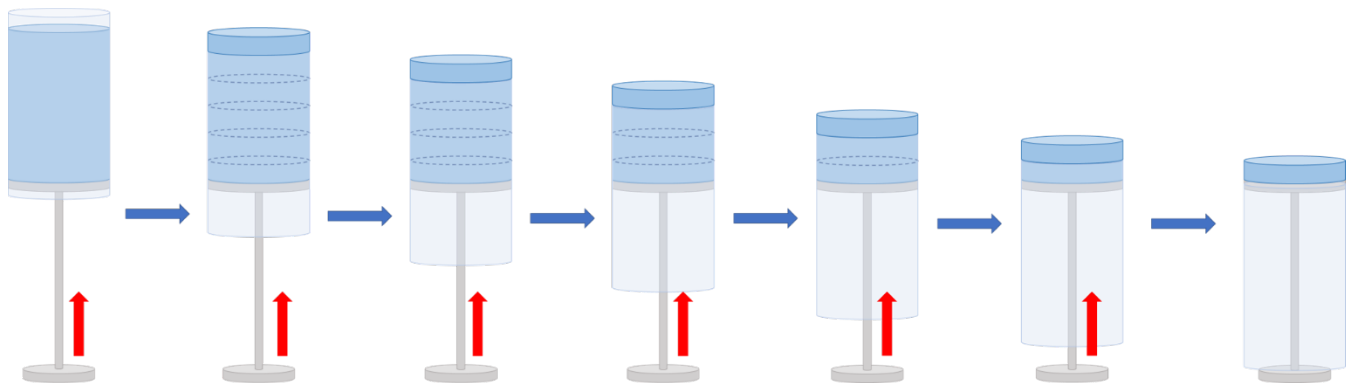

Figure S20. Cartoon showing how the sections of the gels are extruded and cut. Dotted lines are the sections in which the gel will be cut.

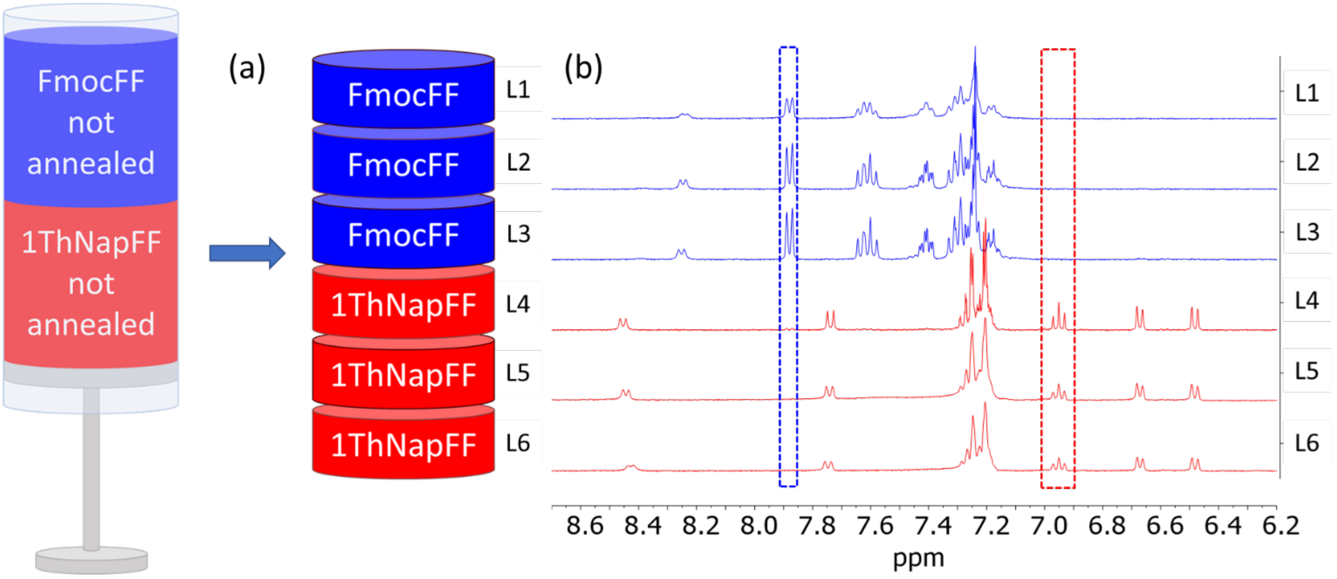

Figure S21. (a) Preparation of gel in syringe for obtaining gel sections. The bottom layer is prepared first with 1ThNapFF in DMSO/H_2O (20/80 v/v), then, after about 45 seconds, the top layer containing FmocFF in DMSO/H_2O (20/80 v/v) is gently poured onto it before gelation occurs. No methyl formate, urea or urease were added in none of the layers. After 16h, the whole system was cut into six sections (L1 to L6). (b) Zoom in the aromatic region (8.7-6.2 ppm) of the spectra of each section, in the blue rectangle the peak chosen for the integration of FmocFF signals, integrating for 2 H, in the red rectangle the peak chosen for the 1ThNapFF signals, integrating for 1 H. In this case, the concentrations of 1ThNapFF and FmocFF are 2 mg/mL and the solvent is DMSO/water (20/80, v/v).

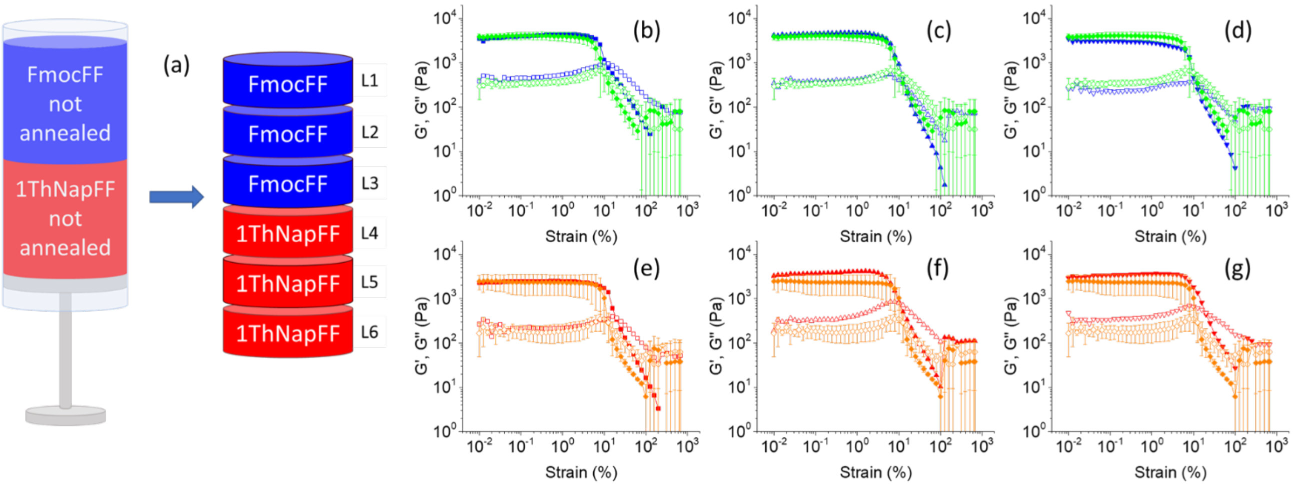

Figure S22. (a) Preparation of gel in syringe for obtaining gel sections. The bottom layer is prepared first with 1ThNapFF in DMSO/H_2O (20/80 v/v), then, after about 45 seconds, the top layer containing FmocFF in DMSO/H_2O (20/80 v/v) is gently poured onto it before gelation occurs. After 16h, the whole system was cut into six

sections (L1 to L6); (b-d) The blue data represent the strain sweeps for the sections L1 to L3 respectively. The green data is the strain sweep for the hydrogel of FmocFF, overlayed as reference to compare. (e-g) The red data represent the strain sweeps for the sections L4 to L6 respectively. The orange data is the strain sweep for the hydrogel of 1ThNapFF, overlayed as reference to compare. For (b-g), all the experiments were carried out using the parallel plate geometry (d = 12.5 mm). The references are the stiffness at $\gamma = 0.05$ % of a single-layer gel of the corresponding peptide prepared, cut and analysed with the same methodology, without annealing. In all cases, the solid symbols represent G′, hollow symbols G″. The concentrations of 1ThNapFF and FmocFF are 2 mg/mL and the solvent is DMSO/water (20/80, v/v) (no methyl formate, urea or urease were used). For each experiment the concentrations of 1ThNapFF and FmocFF are 2 mg/mL and the solvent is DMSO/water (20/80, v/v). All the multilayer experiments have a total volume of about 4 mL, while the reference experiments have a total volume of about 2 mL.

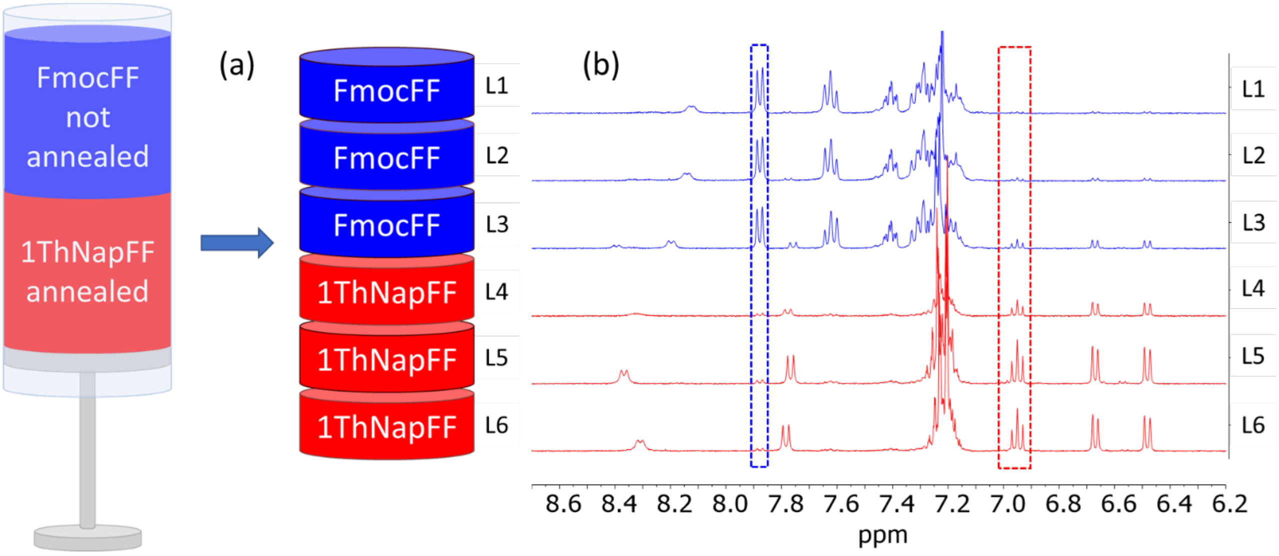

Figure S23. (a) Preparation of gel in syringe for obtaining gel sections. The bottom layer is prepared first with 1ThNapFF in DMSO/H_2O (20/80 v/v), in presence of 0.01 M urea, 0.2 mg/mL urease and 100 µL of methyl formate, then, after about 45 seconds, the top layer containing FmocFF in DMSO/H_2O (20/80 v/v) is gently poured onto it before gelation occurs. After 16h, the whole system was cut into six sections (L1 to L6). (b) Zoom in the aromatic region (8.7-6.2 ppm) of the spectra of each section, in the blue rectangle the peak chosen for the integration of FmocFF signals, integrating for 2 H, in the red rectangle the peak chosen for the 1ThNapFF signals integrating for 1 H. In this case, the concentrations of 1ThNapFF and FmocFF are 2 mg/mL and the solvent is DMSO/water (20/80, v/v). No methyl formate, urea or urease were added in the FmocFF layer, while the 1ThNapFF layer contained 0.01 M urea, 0.2 mg/mL urease and 100 µL of methyl formate were added.

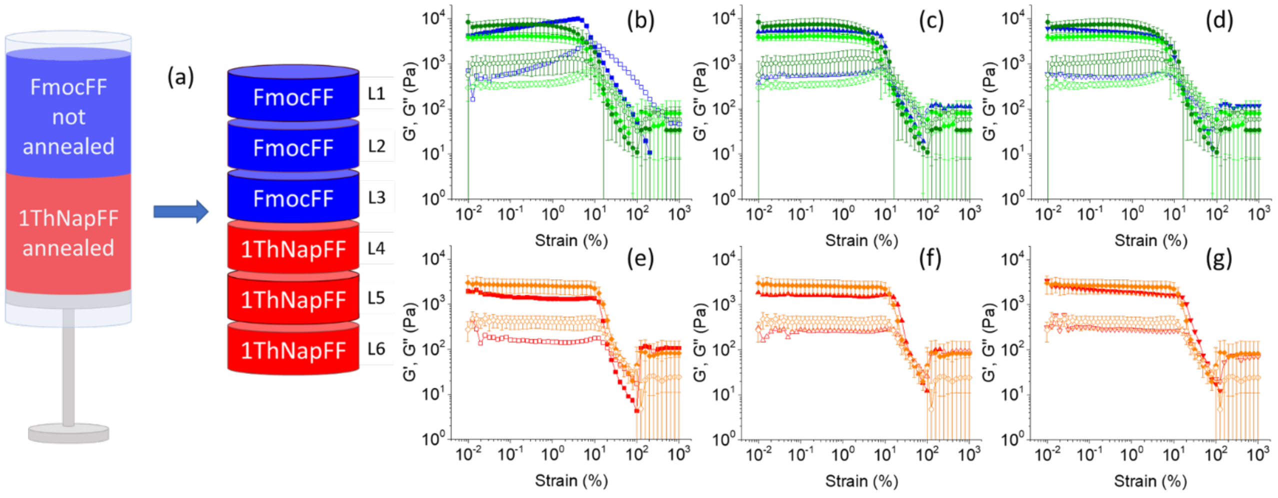

Figure S24. (a) Preparation of gel in syringe for obtaining gel sections. The bottom layer is prepared first with 1ThNapFF in DMSO/H_2O (20/80 v/v), in presence of 0.01 M urea, 0.2 mg/mL urease and 100 µL of methyl formate, then, after about 45 seconds, the top layer containing FmocFF in DMSO/H_2O (20/80 v/v) is gently poured onto it before gelation occurs. After 16h, the whole system was cut into six sections (L1 to L6). (b-d) The blue data represent the strain sweeps for the sections L1 to L3 respectively. The light green and dark green data represent the strain sweeps for FmocFF without and after annealing respectively (overlayed for comparison). (e-g) The red data represent the strain sweeps for the sections L4 to L6 respectively. The orange data represent the strain sweeps for 1ThNapFF obtained after annealing (overlayed for comparison). For (b-g), all the experiments were carried out using the parallel plate geometry (d = 12.5 mm). In all cases, the solid symbols represent G′, hollow symbols G″. The concentrations of 1ThNapFF and FmocFF are 2 mg/mL and the solvent is DMSO/water (20/80, v/v). For annealing the gels, the conditions used are: 0.01 M of urea, 0.2 mg/mL of urease and 100 µL of methyl formate.

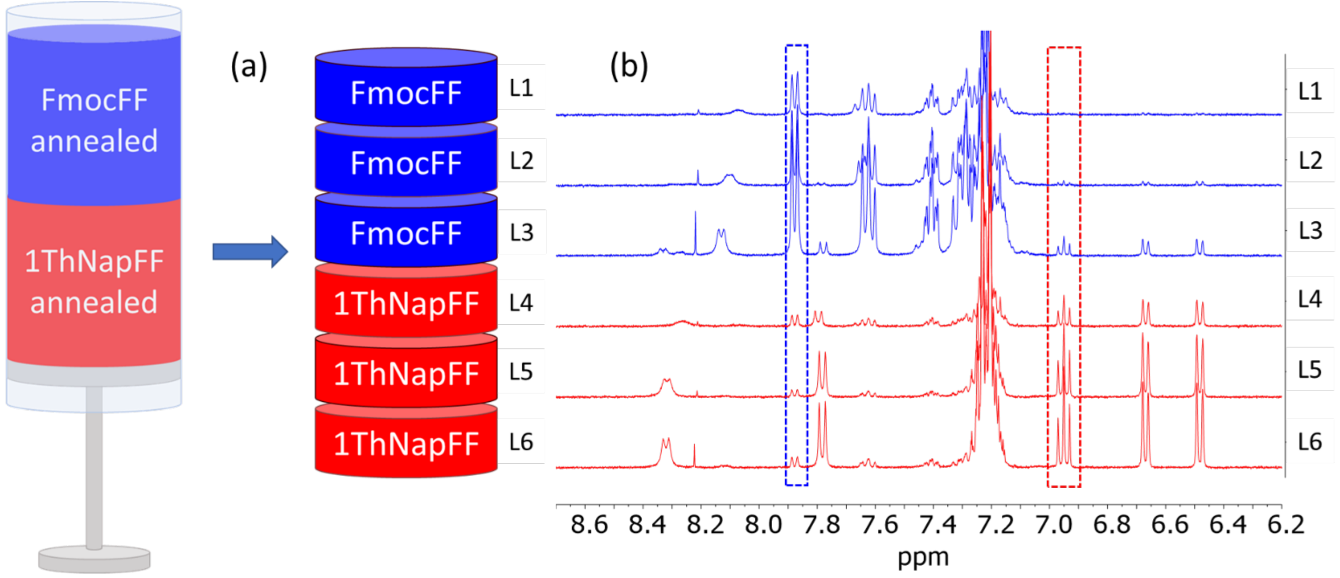

Figure S25. (a) Preparation of gel in syringe for obtaining gel sections. The bottom layer is prepared first with 1ThNapFF in DMSO/H_2O (20/80 v/v), in presence of 0.01 M urea, 0.2 mg/mL urease and 100 µL of methyl formate, then, after about 45 seconds, the top layer containing FmocFF in DMSO/H_2O (20/80 v/v) with 0.01 M urea, 0.2 mg/mL urease and 100 µL of methyl formate is gently poured onto it before gelation occurs. After 16h, the whole system was cut into six sections (L1 to L6). (b) Zoom in the aromatic region (8.7-6.2 ppm) of the spectra of each section, in the blue rectangle the peak chosen for the integration of FmocFF signals, integrating for 2 H, in the red rectangle the peak chosen for the 1ThNapFF signals integrating for 1 H. In this case, the concentrations of 1ThNapFF and

FmocFF are 2 mg/mL and the solvent is DMSO/water (20/80, v/v). In this case in both the layers 0.01 M urea, 0.2 mg/mL urease and 100 µL of methyl formate were added.

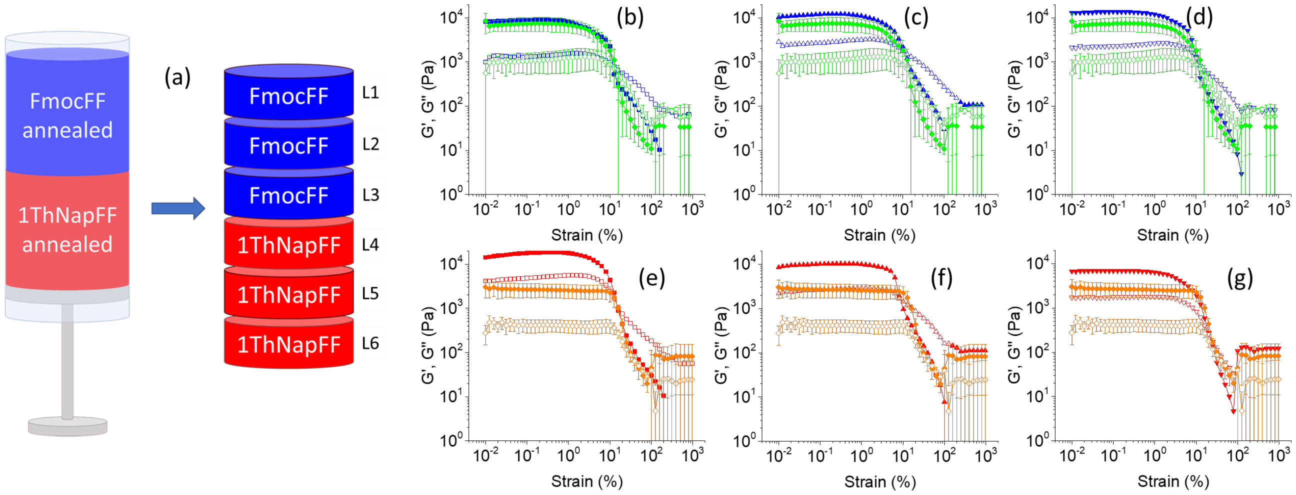

Figure S26. (a) Preparation of gel in syringe for obtaining gel sections. The bottom layer is prepared first with 1ThNapFF in DMSO/H_2O (20/80 v/v), in presence of 0.01 M urea, 0.2 mg/mL urease and 100 µL of methyl formate, then, after about 45 seconds, the top layer containing FmocFF in DMSO/H_2O (20/80 v/v) with 0.01 M urea, 0.2 mg/mL urease and 100 µL of methyl formate is gently poured onto it before gelation occurs. After 16h, the whole system was cut into six sections (L1 to L6). (b-d) The blue data represent the strain sweeps for the sections L1 to L3 respectively. The green data represent the strain sweeps for FmocFF after annealing (overlayed for comparison). (e-g) The red data represent the strain sweeps for the sections L4 to L6 respectively. The orange data represent the strain sweeps for 1ThNapFF obtained after annealing (overlayed for comparison). For (b-g), all the experiments were carried out using the parallel plate geometry (d = 12.5 mm). In all cases, the solid symbols represent G′, hollow symbols G″. The concentrations of 1ThNapFF and FmocFF are 2 mg/mL and the solvent is DMSO/water (20/80, v/v). For annealing the gels, the conditions used are: 0.01 M of urea, 0.2 mg/mL of urease and 100 µL of methyl formate.

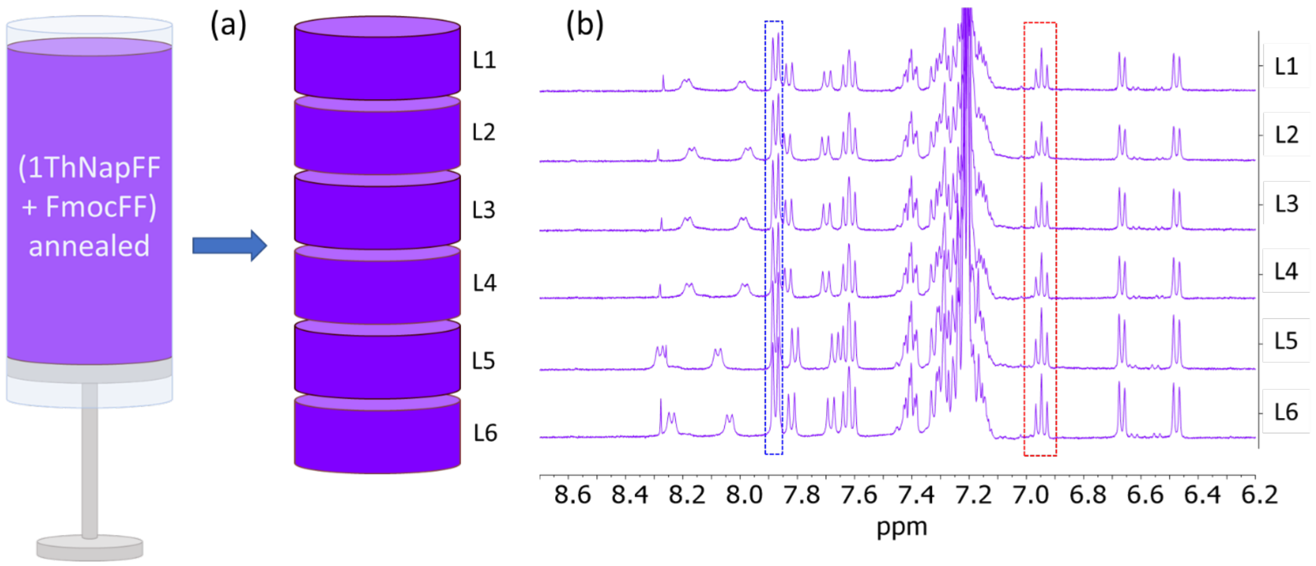

Figure S27. (a) Preparation of gel in syringe for obtaining gel sections. A mixture of 1ThNapFF, FmocFF, urea, urease, methyl formate was prepared first in 4.2 mL total volume. After 16h, the whole system was cut into six sections (L1 to L6). (b) Zoom in the aromatic region (8.7-6.2 ppm) of the spectra of each section, in the blue rectangle the peak chosen for the integration of FmocFF signals, integrating for 2 H, in the red rectangle the peak chosen for the 1ThNapFF signals integrating for 1 H. In this case, the concentrations of 1ThNapFF and FmocFF are 2 mg/mL and the solvent is DMSO/water (20/80, v/v), the concentration of urea is 0.02 M, while urease is 0.4 mg/mL, and the volume of methyl formate added is 200 μL, to obtain a total volume of about 4.2 mL.

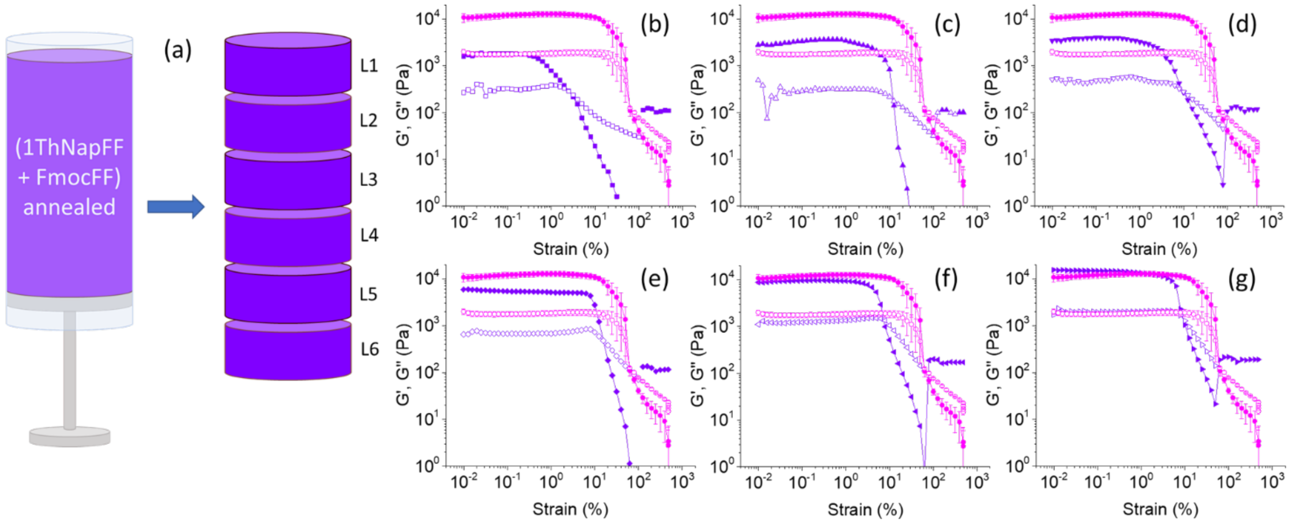

Figure S28. (a) Preparation of gel in syringe for obtaining gel sections. A mixture of 1ThNapFF, FmocFF, urea, urease, methyl formate was prepared first in 4.2 mL total volume. After 16h, the whole system was cut into six sections (L1 to L6). (b-g) The purple data represent the strain sweeps for the sections L1 to L6 respectively measured using the parallel plate geometry (d = 12.5 mm). The pink data in (b-d) is the strain sweep (measured with the cup and vane geometry) of multicomponent gel of (1ThNapFF+ FmocFF) obtained after annealing involving under similar conditions, overlayed as reference for comparison. In all cases, the solid symbols represent G′, hollow symbols G″. The concentrations of 1ThNapFF and FmocFF are 2 mg/mL and the solvent is DMSO/water (20/80, v/v), the concentration of urea is 0.02 M, while urease is 0.4 mg/mL, and the volume of methyl formate added is 200 μL, to obtain a total volume of about 4.2 mL.

References

[1] S. Panja, B. Dietrich, A. Trabold, A. Zydel, A. Qadir, D. J. Adams, *Chem. Commun.* **2021**, *57*, 7898-7901.

[2] J. Raeburn, G. Pont, L. Chen, Y. Cesbron, R. Lévy, D. J. Adams, *Soft Matter* **2012**, *8*, 1168-1174.
